# Isolevuglandins disrupt PU.1-mediated C1q expression and promote autoimmunity and hypertension in systemic lupus erythematosus

**DOI:** 10.1172/jci.insight.136678

**Published:** 2022-07-08

**Authors:** David M. Patrick, Néstor de la Visitación, Jaya Krishnan, Wei Chen, Michelle J. Ormseth, C. Michael Stein, Sean S. Davies, Venkataraman Amarnath, Leslie J. Crofford, Jonathan M. Williams, Shilin Zhao, Charles D. Smart, Sergey Dikalov, Anna Dikalova, Liang Xiao, Justin P. Van Beusecum, Mingfang Ao, Agnes B. Fogo, Annet Kirabo, David G. Harrison

**Affiliations:** 1 Department of Veterans Affairs, Nashville, Tennessee, USA.; 2 Division of Clinical Pharmacology and; 3 Division of Cardiovascular Medicine, Department of Medicine, Vanderbilt University Medical Center, Nashville, Tennessee, USA.; 4 Department of Pharmacology, University of Granada, Granada, Spain.; 5 Division of Rheumatology and Immunology, Department of Medicine, and; 6 Vanderbilt Center for Quantitative Sciences, Vanderbilt University Medical Center, Nashville, Tennessee, USA.; 7 Department of Molecular Physiology and Biophysics,; 8 Ralph H. Johnson VA Medical Center and; 9 Division of Nephrology, Department of Medicine, Medical University of South Carolina, Charleston, South Carolina, USA.; 10 Department of Pathology, Microbiology and Immunology, Vanderbilt University Medical Center, Nashville, Tennessee, USA.

**Keywords:** Autoimmunity, Inflammation, Autoimmune diseases, Dendritic cells, Rheumatology

## Abstract

We describe a mechanism responsible for systemic lupus erythematosus (SLE). In humans with SLE and in 2 SLE murine models, there was marked enrichment of isolevuglandin-adducted proteins (isoLG adducts) in monocytes and dendritic cells. We found that antibodies formed against isoLG adducts in both SLE-prone mice and humans with SLE. In addition, isoLG ligation of the transcription factor PU.1 at a critical DNA binding site markedly reduced transcription of all C1q subunits. Treatment of SLE-prone mice with the specific isoLG scavenger 2-hydroxybenzylamine (2-HOBA) ameliorated parameters of autoimmunity, including plasma cell expansion, circulating IgG levels, and anti-dsDNA antibody titers. 2-HOBA also lowered blood pressure, attenuated renal injury, and reduced inflammatory gene expression uniquely in C1q-expressing dendritic cells. Thus, isoLG adducts play an essential role in the genesis and maintenance of systemic autoimmunity and hypertension in SLE.

## Graphical abstract

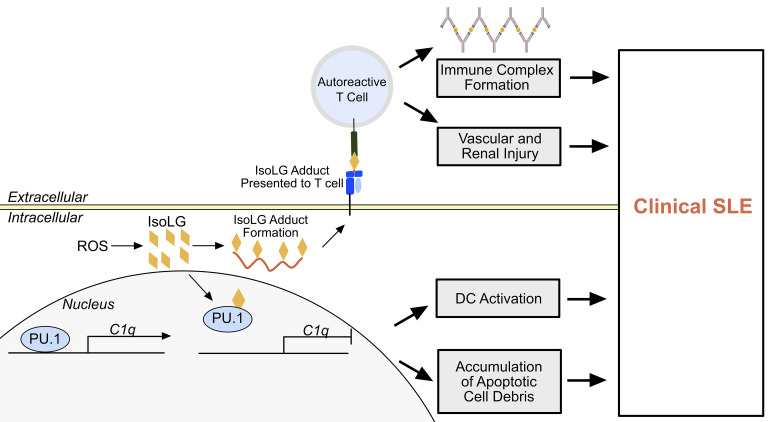

## Introduction

Systemic lupus erythematosus (SLE) is a multiorgan autoimmune disease that affects approximately 9 females for every male (1). SLE is commonly associated with hypertension via mechanisms that are poorly defined (2). Like SLE, immune activation has also been implicated in humans with essential hypertension and in several models of experimental hypertension (3). Interestingly, excessive levels of reactive oxygen species (ROS) have been implicated in both hypertension and SLE. Oxidation-induced apoptosis has been linked to formation of autoantigens in SLE, and autoantibody production is stimulated by oxidative stress (4–8). In the *NZBWF1* mouse model of SLE, treatment at 30 weeks of age with the antioxidant tempol and the NADPH oxidase inhibitor apocynin reduced blood pressure and albuminuria. Such treatment reduces urinary and renal cortical hydrogen peroxide, suggesting that a reduction in oxidative stress attenuates hypertension in SLE (9).

We and others have previously elucidated a role of ROS in the pathogenesis of hypertension. Hypertension is associated with modification of self-proteins by isolevuglandins (isoLGs), which are γ-ketoaldehydes derived from the oxidation of fatty acids and phospholipids (10). IsoLGs react with and covalently modify lysine protein residues. In several mouse models of hypertension, such isoLG adducts accumulate within antigen-presenting cells and promote their maturation and activation (11). Small molecule scavengers of isoLGs, including 2-hydroxybenzylamine (2-HOBA), lower blood pressure and markedly reduce end-organ damage in both angiotensin II (Ang II) and deoxycorticosterone acetate-salt hypertension (11). It is conceivable that isoLG adduction of peptides plays a similar role in SLE; however, this has not been investigated to our knowledge.

C1q is a multiprotein component of the complement system that is composed of C1qA, C1qB, and C1qC chains (12). Deficiency of C1q is strongly associated with SLE. Over 90% of patients with monogenetic mutations in C1q develop an SLE-like syndrome (12, 13). C1q binds to and promotes phagocytosis of apoptotic cellular debris. It has been hypothesized that a defect in phagocytosis of apoptotic cellular debris promotes formation of autoantigens in SLE. Moreover, C1q functions in an autocrine fashion to prevent DC activation (14). Importantly, in both mice and humans, one consensus binding site for the transcription factor PU.1 in the core promoter of *C1qA*, *C1qB*, and *C1qC* genes is responsible for transcription of all C1q subunits (15).

In the present study, we examined monocytes from humans and used 2 mouse models of SLE to define a role of isoLG formation in antigen-presenting cells in the pathogenesis of hypertension and systemic autoimmunity in SLE. Moreover, we identify a potentially novel mechanism of transcriptional repression of C1q by isoLG modification of PU.1.

## Results

### IsoLG adducts are enriched in monocytes of patients with SLE.

**Figure 1:**
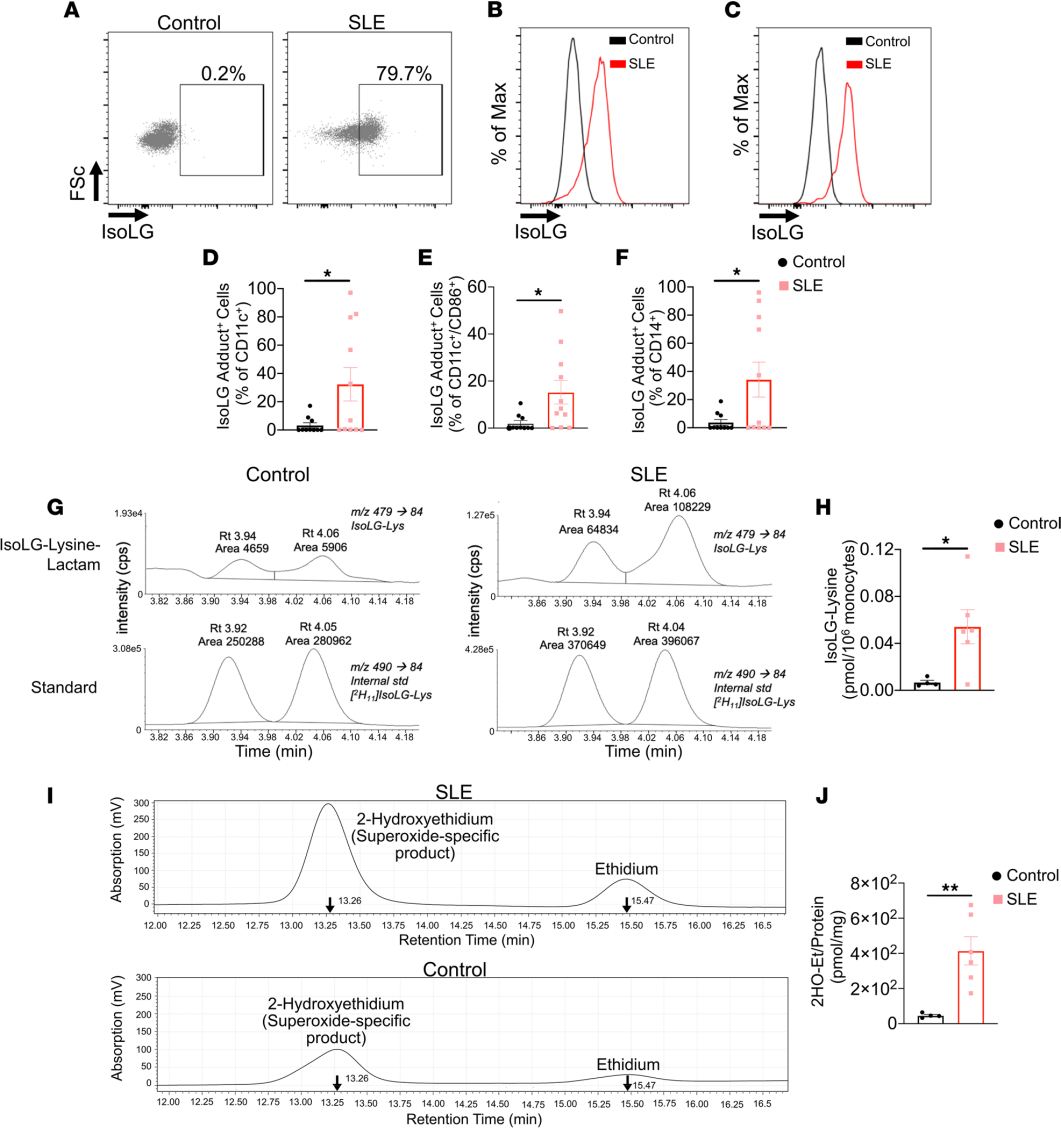
IsoLG adducts are enriched in monocytes of patients with SLE. (**A**) Representative FACS plots displaying isoLG adduct containing CD11c^+^ PBMCs from a representative control and patient with SLE. Representative histograms displaying the distribution of isoLG adducts in (**B**) CD11c^+^ and (**C**) CD11c^+^CD86^+^ cells. Quantitation of IsoLG adduct–containing cells as a percentage of (**D**) CD11c^+^, (**E**) CD11c^+^CD86^+^, and (**F**) CD14^+^ cells. For **B**–**F** data were analyzed using 1-tailed Student’s *t* test or Mann-Whitney *U* test (*n* = 10–11, *P < 0.05). (**G**) Stable isotope dilution multiple reaction monitoring for mass spectrometry analysis of isoLG-lysine-lactam adduct in DCs. Representative liquid chromatography/mass spectrometry chromatographs from a representative patient. The top row shows multiple reaction monitoring chromatographs for isoLG lysine lactam in samples, while the bottom row shows multiple reaction monitoring chromatograph for [13C615N2] internal standard for the same samples. cps, counts per second; Rt, retention time. (**H**) Quantitation of isoLG-lysine in monocytes from a subset of SLE patients and controls. (**I**) Monocytes from SLE patients and controls were sorted. Superoxide was detected using HPLC to monitor conversion of dihydroethidium to the superoxide oxidation adduct 2-hydroxyethidium (2-HO-ET) and ethidium. (**J**) Quantitation of 2-HO-ET from SLE patients and controls. For **H** and **J**, comparisons were made with a 1-tailed Student’s *t* test (*n* = 4–6, *P < 0.05, **P < 0.01).

As an initial attempt to determine if isoLG-modified proteins contribute to the etiology of SLE, we analyzed their presence in circulating monocytes of patients with SLE and matched controls. The majority of patients with SLE were female, and all were positive for antinuclear antibodies. Median disease activity by SLE disease activity index (SLEDAI) was 2 (IQR: 2, 4), 55% had renal involvement, 91% were on hydroxychloroquine, and 55% were on mycophenolate (Supplemental Table 1; supplemental material available online with this article; https://doi.org/10.1172/jci.insight.136678DS1). Additional patient characteristics are shown in Supplemental Table 1. We found that isoLG adducts are enriched in CD11c^+^ and CD14^+^ monocytes and in CD11c^+^ monocytes that are positive for the costimulatory factor CD86 (Figure 1, A–F). In addition to flow cytometry, we used mass spectrometry to quantify isoLG-lysine adducts in monocytes from an additional set of 6 patients with SLE and 4 matched controls. This showed an 8-fold enrichment of isoLG adducts in monocytes of patients with SLE compared with controls (Figure 1, G and H, and Supplemental Table 2). We also saw no relationship between blood pressure and isoLG adducts in monocytes in this cohort (Supplemental Figure 1). Because isoLGs are formed by lipid peroxidation, we also measured monocyte production of superoxide, which can directly oxidize lipids and can yield other ROS, including peroxynitrite, hydroxyl, and hydrogen peroxide, that can promote lipid peroxidation (16). We observed a 9-fold increase in monocyte superoxide production compared with controls (Figure 1, I and J, and Supplemental Table 3). Thus, ROS and isoLG adducts are markedly increased in activated monocytes of humans with SLE.

### B6.SLE123 mice exhibit augmented isoLG adduct accumulation in addition to derangements in immune cell populations.

**Figure 2:**
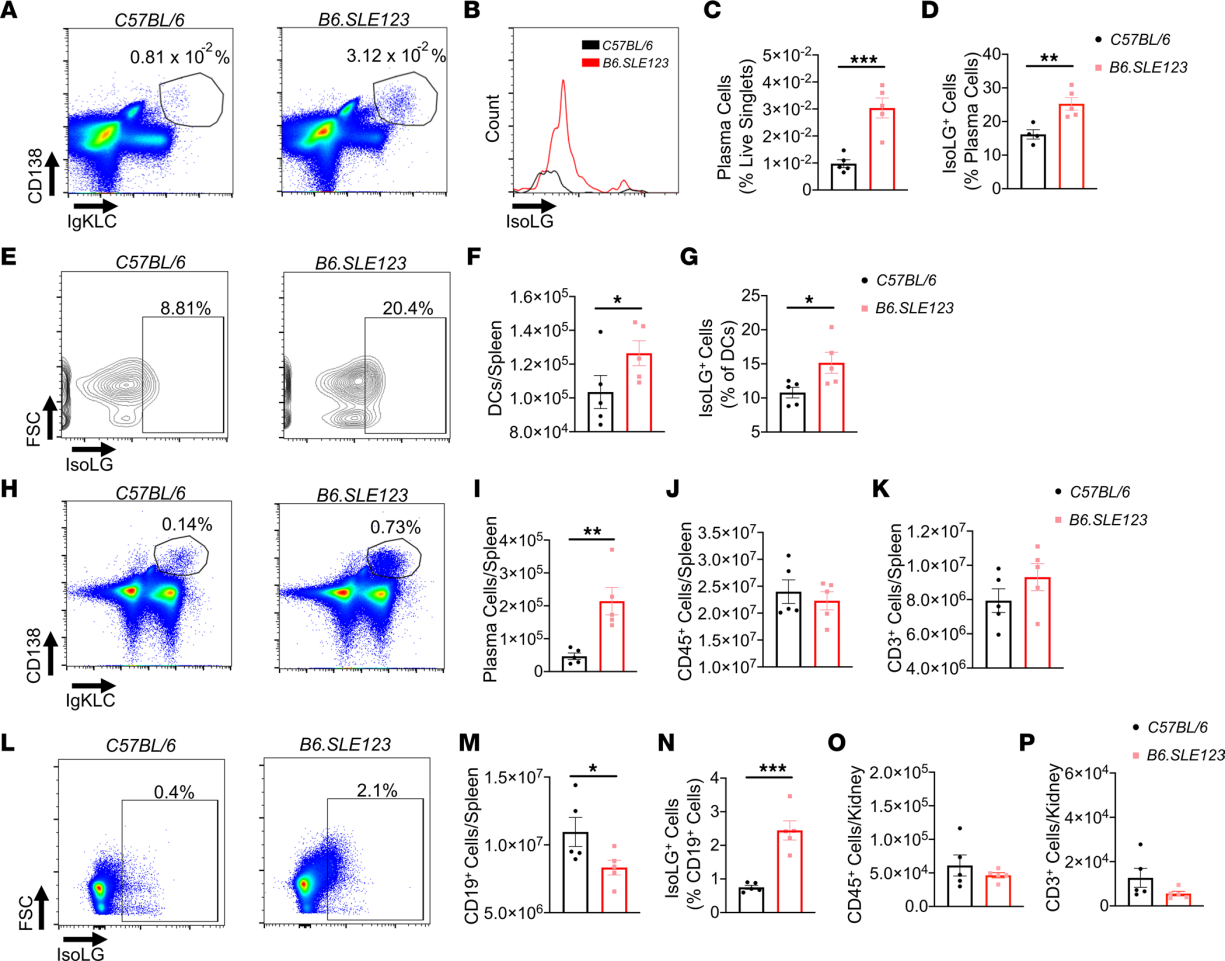
IsoLG adduct accumulation and immune dysfunction in 7-week-old *B6.SLE123* mice. Single-cell suspensions were prepared from freshly isolated mouse tissue via enzymatic digestion and mechanical dissociation. Live cell singlets were analyzed. Representative FACS plots are presented for (**A**) bone marrow plasma cells. (**B**) A histogram of isoLG adduct accumulation in bone marrow plasma cells measured using the single-chain antibody D11 ScFv. (**C**) Quantitation of bone marrow plasma cells. (**D**) IsoLG adduct–containing plasma cells. (**E**) Representative density plots of splenic DC isoLG adduct accumulation. (**F**) Quantitation of DCs in spleen and (**G**) IsoLG adduct–containing DCs in spleen. (**H**) Representative FACS plots of plasma cells in spleen. Quantitation of (**I**) splenic plasma cells, (**J**) splenic CD45^+^ cells, and (**K**) splenic CD3^+^ T cells. (**L**) Representative FACS plots of isoLG adduct accumulation in CD19^+^ B cells in spleen. Quantitation of (**M**) splenic CD19^+^ B cells, (**N**) isoLG adduct–containing CD19^+^ B cells in spleen, (**O**) kidney CD45^+^ cells, and (**P**) kidney CD3^+^ cells. Data were analyzed using 2-tailed Student’s *t* test (*n* = 5, *P < 0.05, **P < 0.01, ***P < 0.001).

To determine if isoLG adduct accumulation precedes the onset of disease, we studied 7-week-old *B6.SLE123* mice. At this age, these animals have not yet developed renal dysfunction, hypertension, or elevation of SLE biomarkers (17). Using flow cytometry and the gating strategies shown in Supplemental Figures 2–4, we found that plasma cells were increased in the bone marrow and observed that isoLG adducts were enriched in these cells in these young *B6.SLE123* female mice (Figure 2, A–D). We also observed an accumulation of DCs in the spleen and increased isoLG adducts in splenic DCs (Figure 2, E–G), and of splenic plasma cells, similar to that observed in the bone marrow (Figure 2, H and I). Total splenic CD45^+^ cells and CD3^+^ T cells were not increased at this age (Figure 2, J and K). B cells from 7-week-old *B6.SLE123* mice exhibited a marked accumulation of isoLG adducts (Figure 2, L–N). Renal immune cell infiltration was not present at this age (Figure 2, O and P). Thus, before the onset of overt disease, there is expansion of bone marrow and splenic antigen-presenting cells that exhibit isoLG adduct accumulation.

### 2-HOBA reduces isoLG adduct accumulation and splenic myeloid/lymphoid expansion in murine SLE.

**Figure 3:**
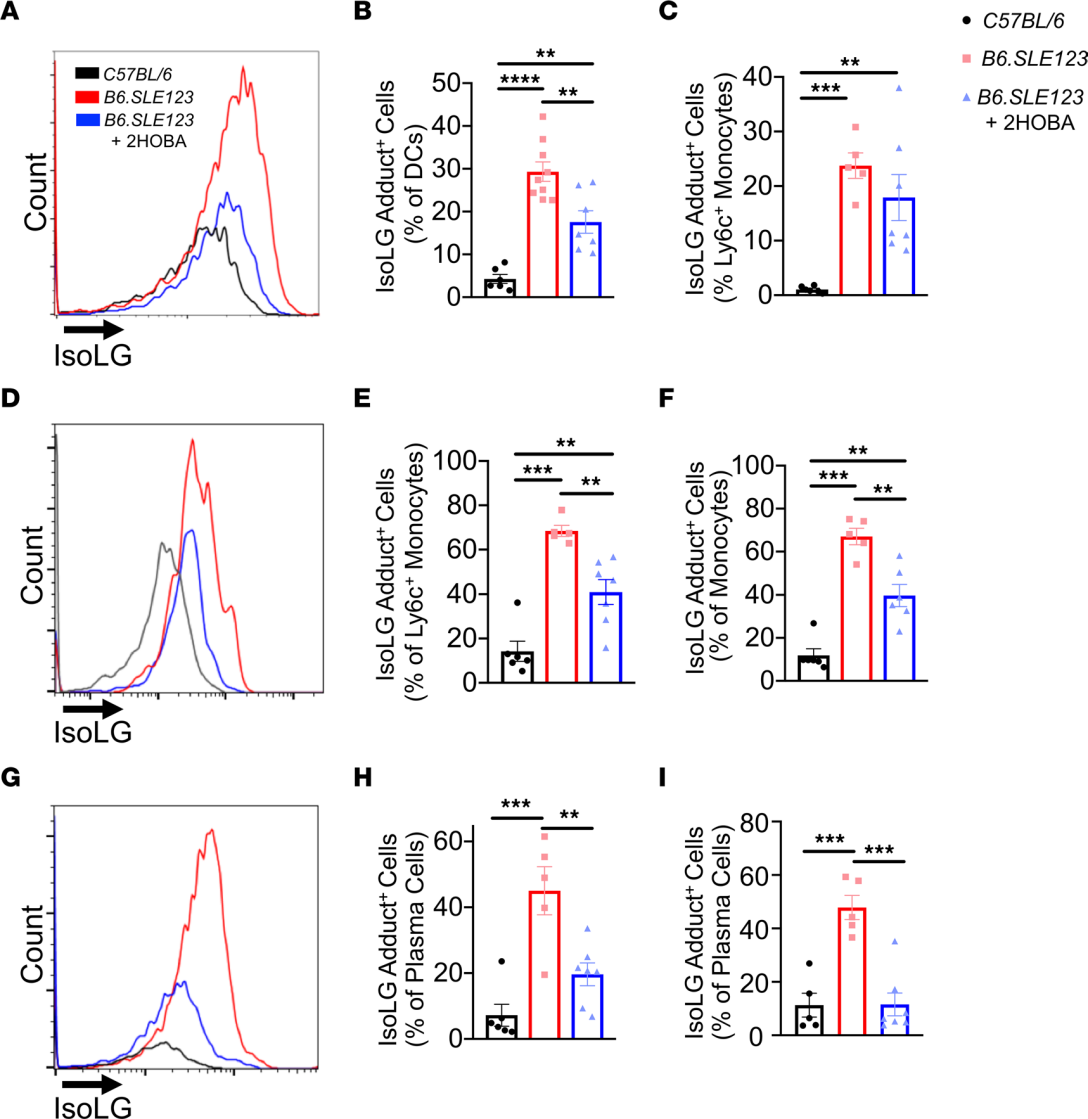
IsoLG adducts are enriched in SLE-prone mice and are efficiently scavenged by 2-HOBA. Cells were isolated at the time of sacrifice from 32-week-old *B6.SLE123* mice. Single-cell suspensions were prepared from freshly isolated mouse tissue via enzymatic digestion and mechanical dissociation. Live cell singlets were analyzed. (**A**) Representative histograms revealing isoLG adduct enrichment and efficient scavenging by 2-HOBA in DCs from spleens. Quantitation of isoLG adduct–containing cells in (**B**) splenic DCs and (**C**) splenic Ly6c^+^ monocytes. (**D**) Representative histogram from peripheral blood Ly6c^+^ monocytes and quantitation of (**E**) isoLG adduct–containing peripheral blood Ly6c^+^ monocytes and (**F**) peripheral blood total monocytes. (**G**) Representative histogram and quantitation of IsoLG adduct–containing (**H**) bone marrow and (**I**) splenic plasma cells. Data were analyzed using 1-way ANOVA (*n* = 5–9, **P < 0.01, ***P < 0.001, ****P < 0.0001).

**Figure 4:**
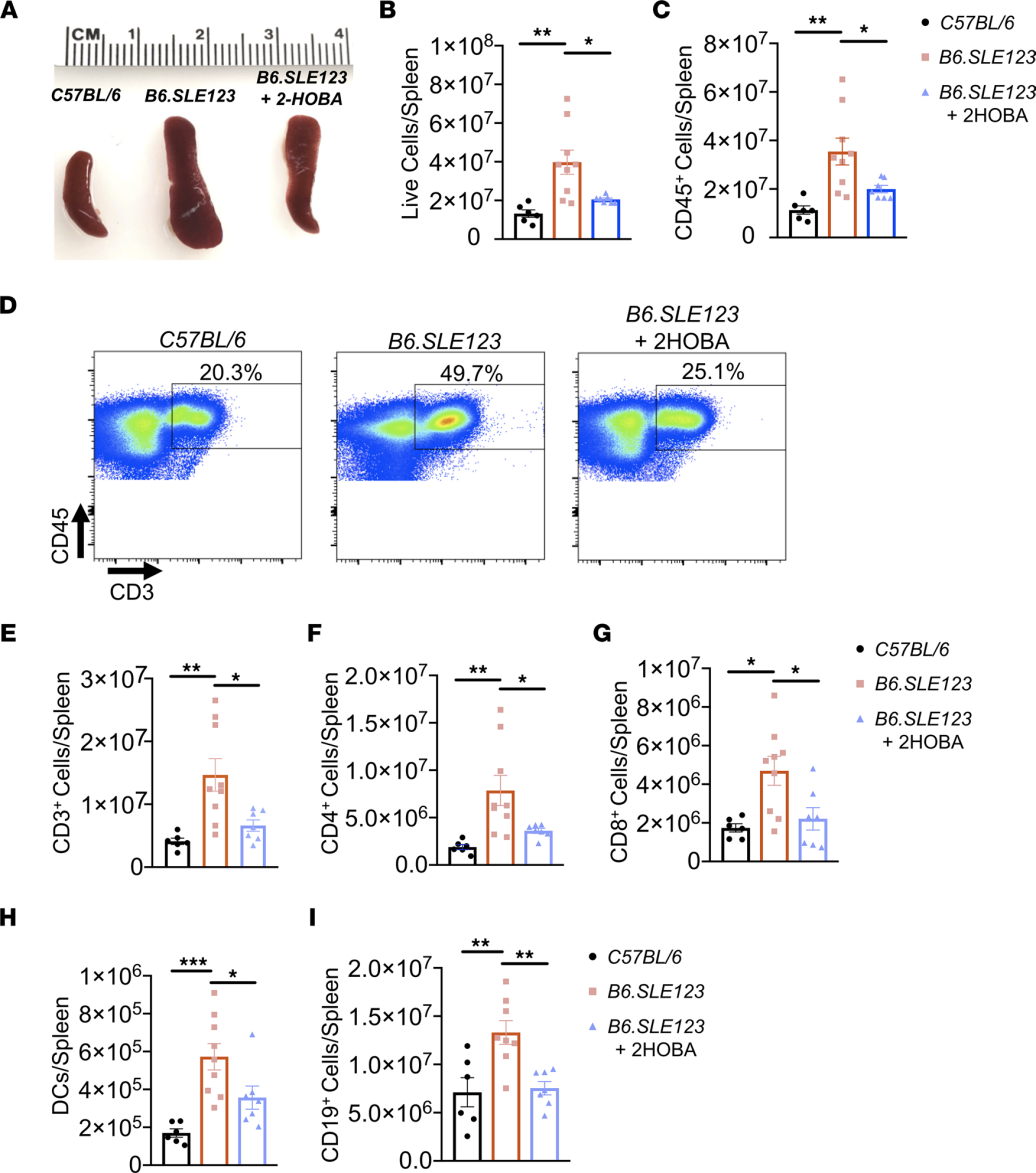
Scavenging of isoLG reduces splenic myeloid and lymphoid expansion in a mouse model of SLE. Spleens and cells were isolated at the time of sacrifice from 32-week-old *B6.SLE123* mice. Single-cell suspensions were prepared from freshly isolated mouse tissue via enzymatic digestion and mechanical dissociation. Live cell singlets were analyzed. (**A**) Representative spleens revealing a reduction in spleen size from *B6.SLE123* mice treated with 2-HOBA. Quantitation of (**B**) live cells and (**C**) CD45^+^ cells. (**D**) Representative FACS plots for CD3^+^ T cells. Quantitation of (**E**) CD3^+^ T cells, (**F**) CD4^+^ T cells, (**G**) CD8^+^ T cells, (**H**) DCs, and (**I**) CD19^+^ B cells. Data were analyzed using 1-way ANOVA (*n* = 6–9, *P < 0.05, **P < 0.01, ***P < 0.001).

To further investigate a role of isoLG adducts in SLE, *B6.SLE123* animals were treated with 2-HOBA from 7 weeks until 32 weeks of age. This agent rapidly forms pyrrole adducts with isoLGs but does not react with superoxide, peroxynitrite, or hydrogen peroxide and exhibits very low reactivity with other lipid oxidation species (11). In *B6.SLE123* mice not treated with 2-HOBA, intracellular isoLG adducts were increased within splenic DCs (Figure 3, A–C), peripheral blood total and Ly6c^+^ monocytes (Figure 3, D–F), bone marrow plasma cells (Figure 3, G and H), and splenic plasma cells (Figure 3I). In addition, we observed an accumulation of isoLG in CD44^hi^ memory B cells compared with naive CD44^lo^ B cells (Supplemental Figure 5, A–C) (18). Treatment with 2-HOBA markedly attenuated the accumulation of isoLG adducts within these cells. *B6.SLE123* mice exhibited a marked increase in spleen size and an expansion of myeloid and lymphoid cells compared with *C57BL/6* mice, and 2-HOBA caused a marked reduction in spleen size in the lupus-prone mice (Figure 4A). Reduction in spleen size was accompanied by a decrease in total splenic cell number (Figure 4B), fewer CD45^+^ cells (Figure 4C), and fewer DCs and CD3^+^, CD4^+^, CD8^+^, and CD19^+^ cells (Figure 4, D–I). Thus, 2-HOBA ameliorates isoLG adduct accumulation and the expansion of multiple cell types in SLE.

### SLE-associated hypertension, renal dysfunction, inflammation, and immune complex–mediated renal injury are attenuated by 2-HOBA.

**Figure 5:**
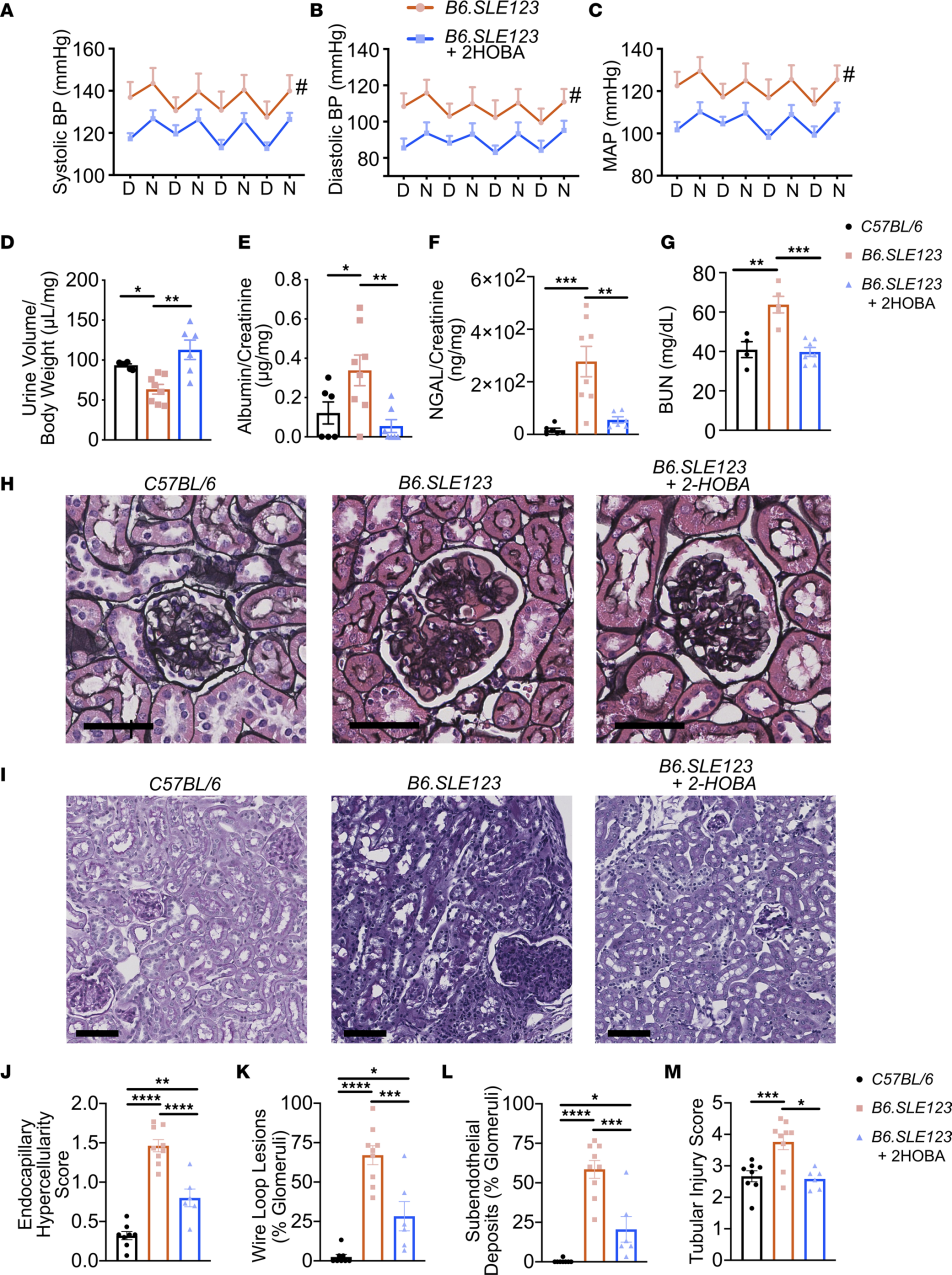
IsoLG scavenging attenuates hypertension and renal injury in a mouse model of SLE. Radiotelemeters were implanted in 30-week-old animals. Measurements were made prior to sacrifice at 32 weeks old. Average measurements over a 4-day period. Day (D) and night (N) cycles are represented for (**A**) systolic, (**B**) diastolic, and (**C**) mean arterial pressures. Blood pressure was analyzed using 2-way ANOVA (*n* = 6–7, ^#^*P* < 0.001). Urine studies were performed on 31-week-old animals. (**D**) Mice received intraperitoneal injection of 4% normal saline at 10% of body weight and urine output was measured after 4 hours. (**E**) Spot urine albumin/creatine ratio. (**F**) Spot urinary NGAL, which represents tubular injury. (**G**) Plasma BUN was quantitated. Animals were sacrificed at 32 weeks old, and kidneys were sectioned and stained with Jones’ silver stain. (**H**) Representative glomeruli are presented (scale bar = 40 μm). Kidneys were also stained with PAS (**I**) Representative sections showing tubular structure (scale bar = 80 μm). Kidneys were scored for (**J**) severity of endocapillary hypercellularity, (**K**) presence of wire loop lesions, (**L**) subendothelial deposits, and (**M**) tubular injury. Data were analyzed by 1-way ANOVA (*n* = 6–9, *P < 0.05, **P < 0.01, ***P < 0.001, ****P < 0.0001).

Using telemetry, *B6.SLE123* mice were found to develop significant hypertension at 32 weeks of age. Long-term treatment with 2-HOBA significantly reduced systolic, diastolic, and mean arterial blood pressures in *B6.SLE123* animals when compared with *B6.SLE123* animals treated with vehicle (Figure 5, A–C).

Normally, mice excrete about 90% of an acute volume challenge within 4 hours, and this response is impaired in several models of experimental hypertension (19, 20). After injection of isotonic saline equal to 10% body weight, urine production was significantly reduced in *B6.SLE123* animals compared with healthy controls (19, 21, 22) but was normalized by treatment with 2-HOBA (Figure 5D). The albumin-to-creatinine ratio, urinary excretion of neutrophil gelatinase-associated lipocalin (NGAL), and plasma blood urea nitrogen (BUN) were significantly elevated in *B6.SLE123* animals and were likewise improved with 2-HOBA treatment (Figure 5, E–G). Jones’ silver stain of paraffin-embedded kidneys revealed that 2-HOBA treatment also reduced glomerular hypercellularity, wire loop lesions, and immune complex deposition (Figure 5, H and J–L). Consistent with a reduction in urinary NGAL, Periodic acid–Schiff (PAS) staining revealed improvement in renal tubular injury with 2-HOBA treatment (Figure 5, I, L, and M). These findings suggest that isoLGs contribute to renal inflammation, injury, and immune complex deposition in SLE.

**Figure 6:**
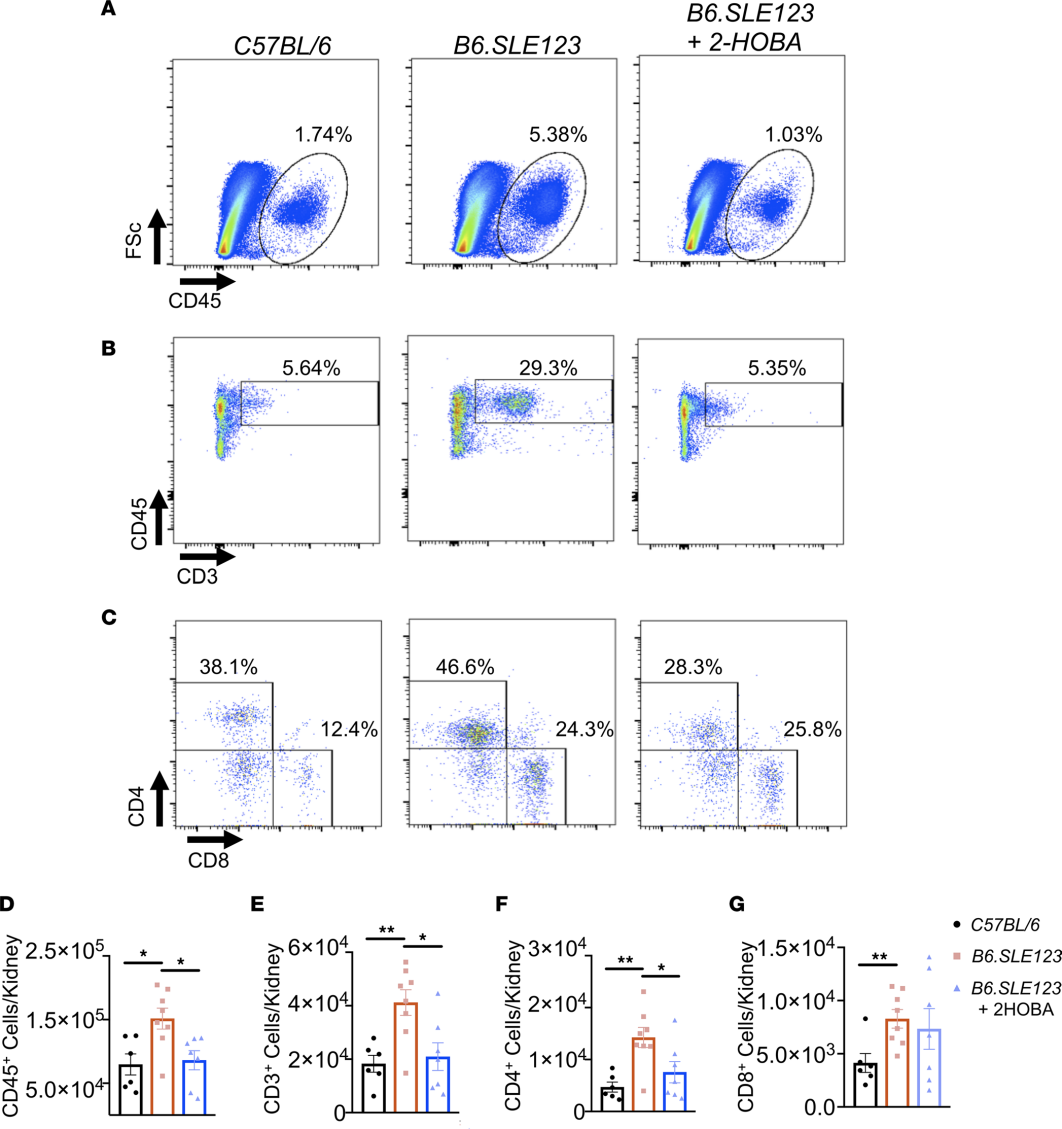
IsoLG scavenging attenuates renal inflammation in a mouse model of SLE. Cells were isolated at the time of sacrifice from 32-week-old *B6.SLE123* mice. Single-cell suspensions were prepared from freshly isolated mouse tissue via enzymatic digestion and mechanical dissociation. Live cell singlets were analyzed. Representative FACS plots are presented for (**A**) CD45^+^ total leukocytes, (**B**) CD3^+^ T cells, and (**C**) CD4^+^ and CD8^+^ T cells. Quantitation of (**D**) CD45^+^ leukocytes, (**E**) CD3^+^ T cells, and (**F**) CD4^+^ and (**G**) CD8^+^ T cells. Data were analyzed using 1-way ANOVA (*n* = 6–8, *P < 0.05, **P < 0.01).

Moreover, we observed a marked reduction in renal CD45^+^ cells, CD3^+^ T cells, and CD4^+^ T cells in *B6.SLE123* animals treated with 2-HOBA (Figure 6, A–F). The presence of CD8^+^ T cells, F4/80^+^ macrophages, or Ly6c^+^ monocytes in the kidney was not affected by 2-HOBA (Figure 6G and Supplemental Figure 6). The percentage of renal isoLG^+^CD45^+^ cells was significantly lower (*P* < 0.05) than that observed in spleen, blood, and bone marrow. Importantly, the level of isoLG adducts was markedly lower in CD45^–^ cells compared with CD45^+^ cells within the kidney (Supplemental Figure 7), indicating that the bone marrow–derived cells predominantly accumulated these adducts. Thus, isoLG scavenging markedly reduces blood pressure and renal inflammation in this model of SLE.

### 2-HOBA reduces plasma cell expansion, autoantibody production, and anti-isoLG antibody production.

**Figure 7:**
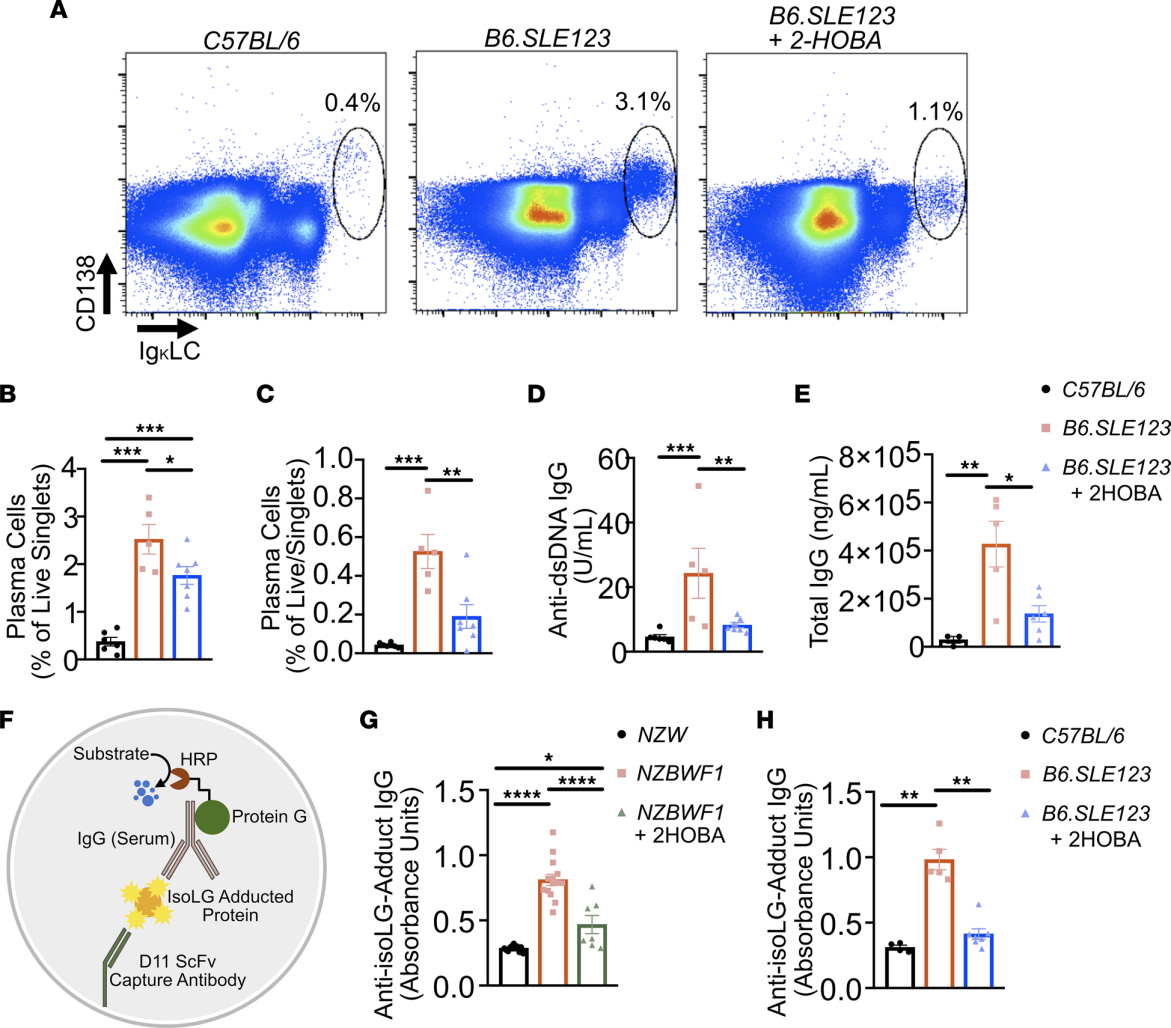
Plasma cell accumulation, autoantibody production, and anti-isoLG IgG levels are attenuated by 2-HOBA in a mouse model of SLE. Cells were isolated from 32-week-old *B6.SLE123* mice. Single-cell suspensions were prepared from freshly isolated mouse tissue via enzymatic digestion and mechanical dissociation. Live cell singlets were analyzed. (**A**) Representative FACS plots displaying CD138^+^ and intracellular Ig κ light chain^+^ plasma cells from bone marrow. Quantitation of plasma cells as a percentage of live singlet cells for (**B**) spleen and (**C**) bone marrow. (**D**) Anti-dsDNA IgG antibody and (**E**) total IgG at 1:100,000 dilution were quantified from plasma using ELISA. Data were analyzed by 1-way ANOVA (*n* = 5–8, *P < 0.05, **P < 0.01, ***P < 0.001, ****P < 0.0001). Serum was collected from mice at the time of sacrifice at 32 weeks old. Antibodies against isoLG adducts were determined by ELISA from serum. (**F**) Model of capture assay to detect anti-isoLG adduct IgG. The D11 ScFv single-chain antibody was bound to a plate. Kidney protein was adducted with isoLG in vitro and incubated with D11 ScFv. Serum from lupus-prone mice was then added, and after extensive washing, the presence of bound IgG was detected utilizing a protein-G HRP conjugate. Anti-isoLG adduct IgG was detected in (**G**) *NZBWF1* mice treated with vehicle or 2-HOBA compared with *NZW* controls and (**H**) *B6.SLE123* mice treated with vehicle or 2-HOBA compared with *C57BL/6* controls. Data were analyzed with 1-way ANOVA with Tukey’s post hoc test (*n* = 4–7 for *B6.SLE123*, *n* = 7–14 for *NZBWF1*; *P < 0.05, **P < 0.01, ****P < 0.0001).

Flow cytometry revealed a significant accumulation of spleen and bone marrow plasma cells in *B6.SLE123* animals, which was attenuated by treatment with 2-HOBA (Figure 7, A–C). Plasma cells are responsible for secretion of autoantibodies in SLE. We found that *B6.SLE123* animals treated with 2-HOBA exhibited markedly lower plasma anti-dsDNA antibody and total IgG antibody titers (Figure 7, D and E). These data demonstrate that scavenging of isoLGs with 2-HOBA attenuates plasma cell accumulation and autoantibody production.

Previous studies have described the autoantigenicity of 4-oxo-2-nonenal– and 4-hydroxy-2-nonenal–adducted peptides in SLE (23). We hypothesized that isoLG-adducted protein would react with serum from mice with SLE. Using a capture ELISA, we found that both *B6.SLE123* and *NZBWF1* mice exhibited increased isoLG adduct-specific IgG antibodies (Figure 7F). Importantly, scavenging of isoLG with 2-HOBA prevented accumulation of these antibodies (Figure 7, G and H).

### Anti-isoLG antibodies correlate with SLEDAI in human participants.

**Figure 8:**
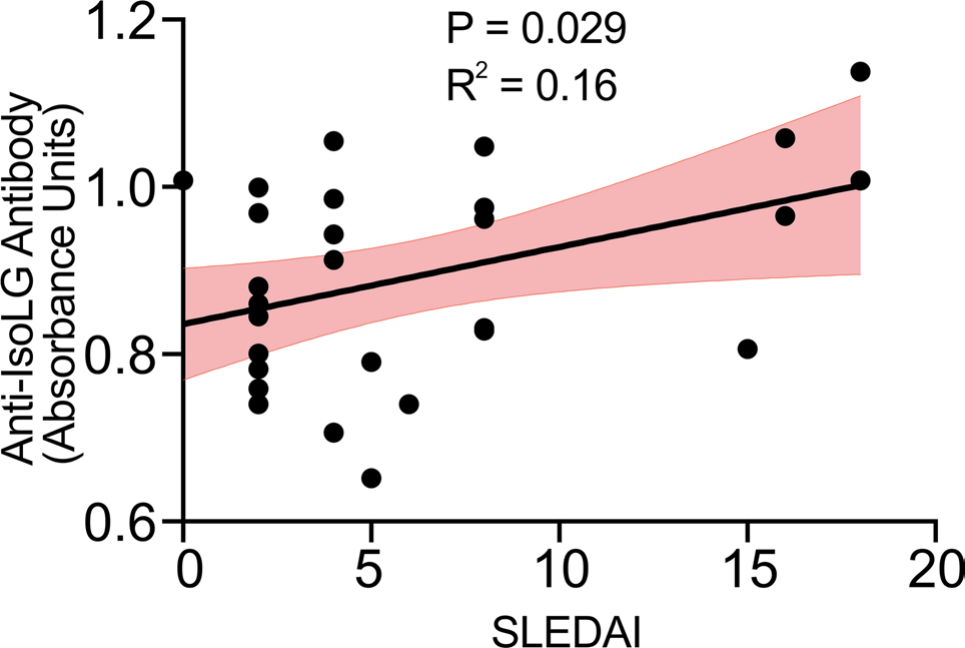
Anti-isoLG IgG in plasma from human participants with SLE positively correlates with SLEDAI. Data were analyzed by Pearson correlation. The 95% confidence interval is represented in red (*n* = 29).

Using the anti-isoLG antibody capture ELISA, we compared plasma reactivity to isoLG adducts with SLEDAI in plasma samples from 29 additional patients with SLE (Supplemental Table 4). Median disease activity by SLEDAI was 4 (IQR: 2, 8). We observed a positive correlation of reactivity to isoLG adducts with SLEDAI (Figure 8).

### 2-HOBA reduces blood pressure, renal inflammation, and plasma cell accumulation in the NZBWF1 mouse model of SLE.

**Figure 9:**
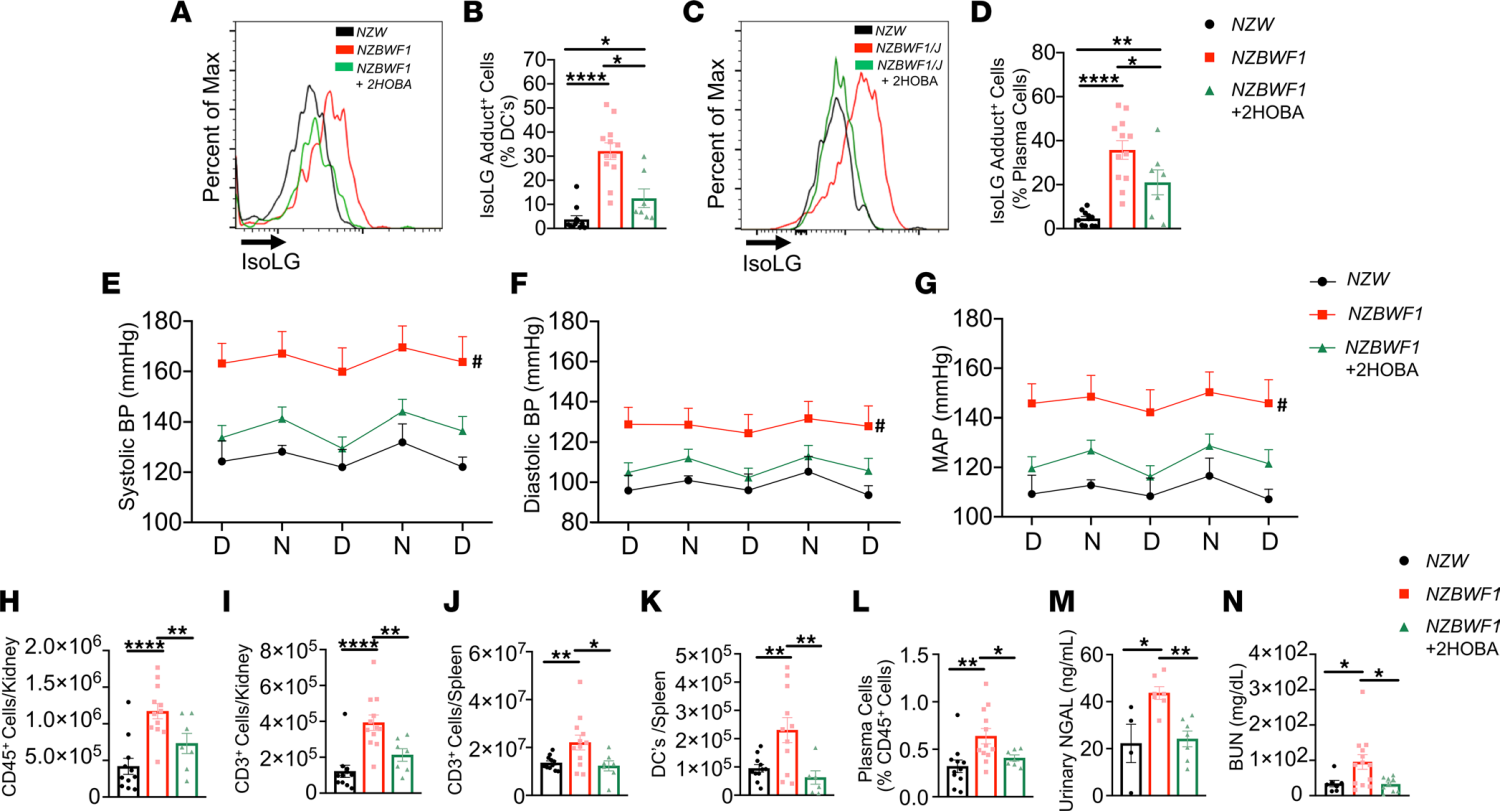
IsoLG scavenging reduces blood pressure and systemic autoimmunity in the *NZBWF1* mouse model of SLE. Animals were sacrificed at 32 weeks, and single-cell suspensions were prepared from the spleen, kidney, and bone marrow and analyzed by flow cytometry. (**A**) Representative histogram displaying the distribution of IsoLG adduct–containing splenic DCs. (**B**) Quantitation of IsoLG adduct–containing splenic DCs. (**C**) Representative histogram displaying the distribution of IsoLG adduct–containing bone marrow plasma cells. (**D**) Quantitation of IsoLG adduct–containing bone marrow plasma cells. Radiotelemeters were implanted in 30-week-old mice. Blood pressure was measured over a 3-day period prior to sacrifice at 32 weeks old. Day and night cycles are represented for (**E**) systolic, (**F**) diastolic, and (**G**) mean arterial pressures. Flow cytometry was performed for quantitation of (**H**) kidney CD45^+^ and (**I**) kidney CD3^+^ T cells, (**J**) splenic CD3^+^ T cells, (**K**) splenic DCs, and (**L**) bone marrow plasma cells. (**M**) Urinary NGAL and (**N**) BUN were quantitated by ELISA. Data from **B**, **D**, and **H**–**M** were analyzed by 1-way ANOVA or Brown-Forsythe and Welch ANOVA (*n* = 7–12, *P < 0.05, **P < 0.01, ****P < 0.0001). Blood pressure was analyzed using 2-way ANOVA (*n* = 5–9, ^#^*P* < 0.001 *NZBWF1* vs. *NZBWF1* + 2-HOBA).

We confirmed the role of isoLG adducts in the *NZBWF1* mouse model of SLE. Untreated *NZBWF1* animals displayed a marked accumulation of isoLG adducts within splenic DCs and bone marrow plasma cells, similar to that observed in the *B6.SLE123* model, and 2-HOBA efficiently prevented this (Figure 9, A–D). In this model, we also found that 2-HOBA lowered systolic, diastolic, and mean arterial pressures (Figure 9, E–G), as measured by radiotelemetry. Moreover, renal immune cell and T cell infiltration was attenuated by 2-HOBA treatment, similar to the *B6.SLE123* model (Figure 9, H and I). Total splenic T cells and DCs (Figure 9, J and K) and bone marrow plasma cell accumulation were reduced with 2-HOBA treatment (Figure 9L). Similar to what we observed in the *B6.SLE123* model, 2-HOBA treatment reduced urinary NGAL excretion and plasma BUN in *NZBWF1* mice (Figure 9, M and N). These data validate the findings observed in the *B6.SLE123* in an independent model of SLE.

### C1q expression is increased by 2-HOBA treatment in SLE-prone mice, and isoLG adduction reduces PU.1 binding to C1q promoter.

**Figure 10:**
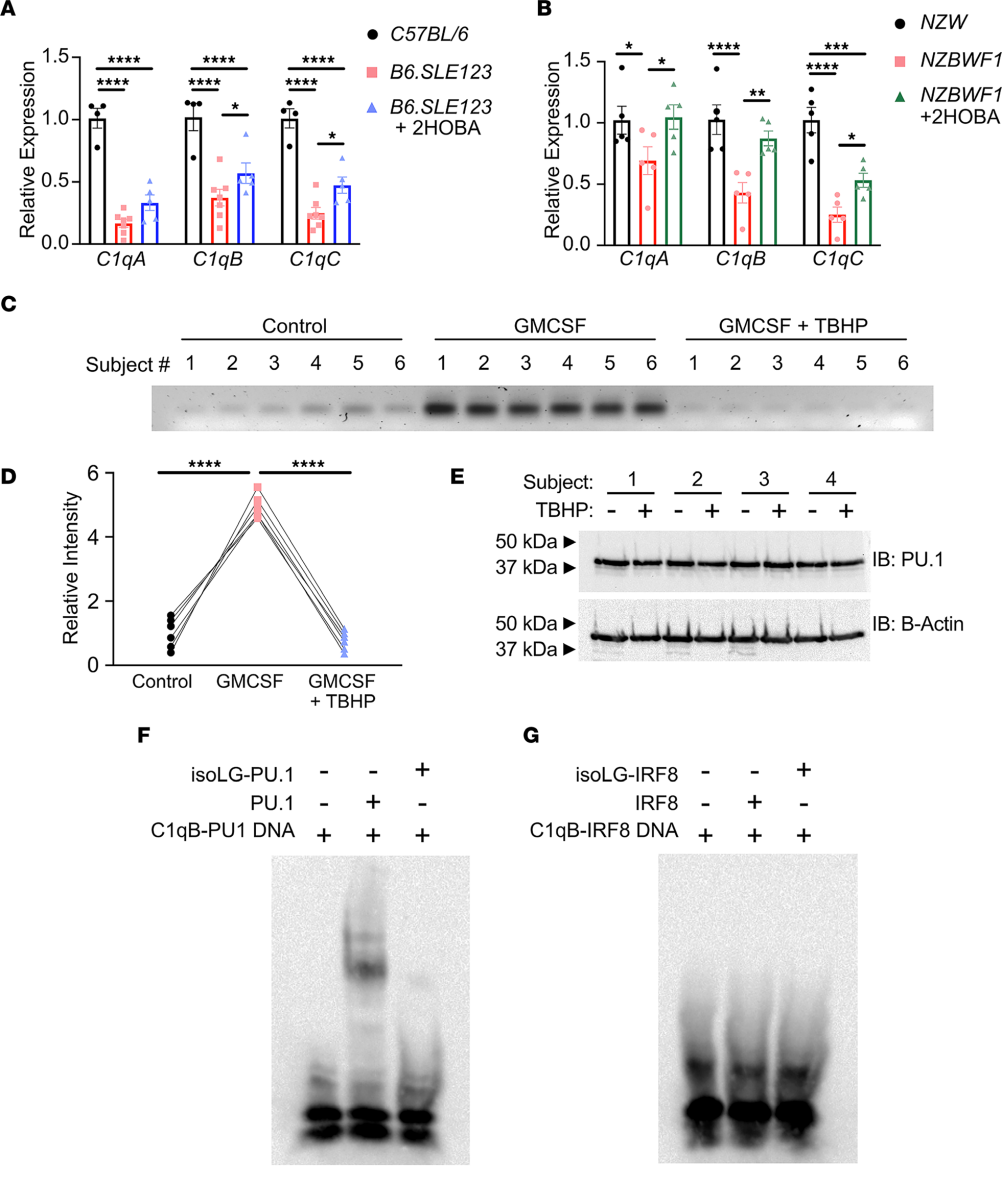
IsoLG adduction of PU.1 disrupts binding to C1q. Animals were treated with 2-HOBA beginning at 7 weeks of age and sacrificed after 6 weeks of treatment. DCs were harvested and analyzed by RT-PCR. Quantitation of *C1qA*, *C1qB*, and *C1qC* transcripts in (**A**) *B6.SLE123* mice treated with vehicle or 2-HOBA compared with *C57BL/6* controls and (**B**) *NZBWF1* mice treated with vehicle or 2-HOBA compared with *NZW* controls. Data were analyzed with 1-way ANOVA with Tukey’s post hoc test (*n* = 4–5). (**C**) Human monocytes were harvested from 6 healthy controls and cultured in the presence of vehicle, GM-CSF, or GM-CSF with a 30-minute exposure to *tert*-butyl-hydroperoxide (tBHP). Chromatin fragments were incubated with PU.1 antibody on a Sepharose substrate. Precipitated chromatin was eluted, and PCR was performed on extracted DNA. PCR was examined on a 2% agarose gel. (**D**) Quantitation of band intensity is represented. Data were analyzed with a repeated measures 1-way ANOVA with Tukey’s post hoc test (*n* = 6). *P < 0.05, **P < 0.01, ***P < 0.001, ****P < 0.0001. (**E**) Human monocytes were cultured in the presence of GM-CSF with or without a 30-minute exposure to tBHP. Protein lysate was analyzed for PU.1 expression by immunoblot. (**F**) A 3′ biotin-labeled annealed DNA fragment containing the C1q PU.1 binding site was incubated in the presence of absence of PU.1 or previously isoLG-adducted PU.1. (**G**) A 3′ biotin-labeled annealed DNA fragment containing the C1q IRF8 binding site was incubated in the presence or absence of IRF8 or previously isoLG-adducted IRF8. Binding reactions were examined on a Tris-borate-EDTA acrylamide gel and imaged on a nylon membrane.

We next performed RNA sequencing of the entire transcriptome in DCs from 7-week-old female *B6.SLE123* and *C57BL/6* controls. At an adjusted *P* value of less than 0.05, we found 7777 differentially expressed genes between *B6.SLE123* and *C57BL/6* mice, indicating large-scale perturbations in the transcriptome. Importantly, Kyoto Encyclopedia of Genes and Genomes (KEGG) pathway analysis revealed differential expression of complement and coagulation cascade transcripts that was notable for marked downregulation of early components of the complement system, including *C1qa*, *C1qb*, and *C1qc* (Supplemental Table 5). Downregulation of C1q transcripts in DCs was validated by real-time reverse transcription PCR (RT-PCR) in both *B6.SLE123* and *NZBWF1* mouse models of SLE. Importantly, a 6-week treatment of 2-HOBA partially restored expression of C1q subunit transcripts, suggesting that C1q expression within DCs is regulated by isoLG formation (Figure 10, A and B).

The PU.1 site within the core C1q promoter is located within the *C1qb* locus and is highly conserved across 60 vertebrate species, including between mice and humans (Supplemental Figure 8). Chromatin immunoprecipitation (ChIP) revealed significant interaction (*P* < 0.0001) of PU.1 with the C1q locus in human monocytes treated with GM-CSF, a known activator of PU.1-mediated transcription (24). Exposure of human monocytes to *tert*-butyl-hydroperoxide (tBHP), which induces intracellular isoLG formation, markedly reduced PU.1 binding to this locus (Figure 10, C and D) (11). Cotreatment of monocytes with GM-CSF and tBHP did not change PU.1 expression (Figure 10E). Importantly, in an electrophoretic mobility shift assay (EMSA), direct isoLG adduction of the PU.1 transcription factor abrogates binding to the *C1q* consensus site (Figure 10F). We did not observe binding of adducted or unadducted interferon regulatory factor 8 (IRF8) to the previously reported consensus sequence located within the *C1q* core promoter (Figure 10G).

### Intracellular isoLG scavenging restores tBHP-mediated repression of PU.1 activity.

**Figure 11:**
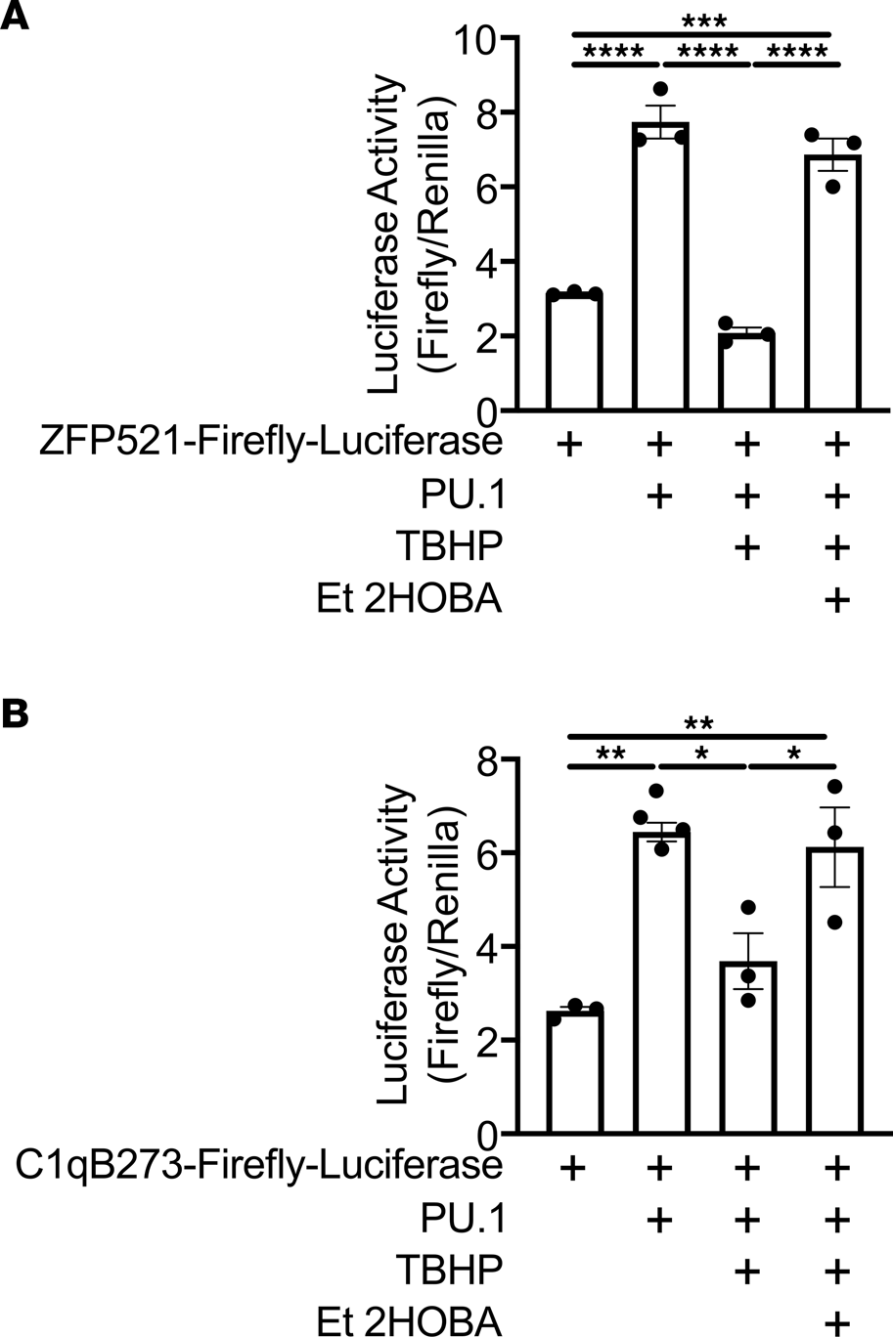
TBHP-mediated repression of PU.1 transcriptional activity is restored with Et-2-HOBA. HEK293T cells were transfected with (**A**) ZFP521-Luciferase or (**B**) C1qB273-Luciferase and cotransfected with a PU.1 expression vector. Cells were then treated with tBHP or cotreated with tBHP and Et-2-HOBA. SV40-Renilla-Luciferase construct was used as an expression control. Data were analyzed by 1-way ANOVA with Tukey’s post hoc test (*n* = 3–4, *P < 0.05, **P < 0.01, ***P < 0.001, ****P < 0.0001).

To determine the direct effect of isoLG adduction on PU.1 transcriptional activity, we obtained the ZFP521-Luciferase reporter vector and PU.1 expression construct previously described (25). We also generated the PU.1-responsive C1qB273 luciferase reporter vector as previously described (15). HEK293T cells were cotransfected with PU.1 and ZFP521-Luciferase or C1qB273-Luciferase. Exposure to tBHP repressed transcriptional activation of both reporters. Cells were also cotreated with tBHP and Et-2-HOBA, a highly cell-permeable analog of 2-HOBA and specific isoLG scavenger (26, 27). Cotreatment with Et-2-HOBA restored PU.1-mediated transcriptional activity (Figure 11, A and B). Thus, tBHP repression of PU.1 activity is mediated by isoLG.

### Mutation of PU.1 lysine 228 prevents tBHP-mediated repression of C1q activation.

**Figure 12:**
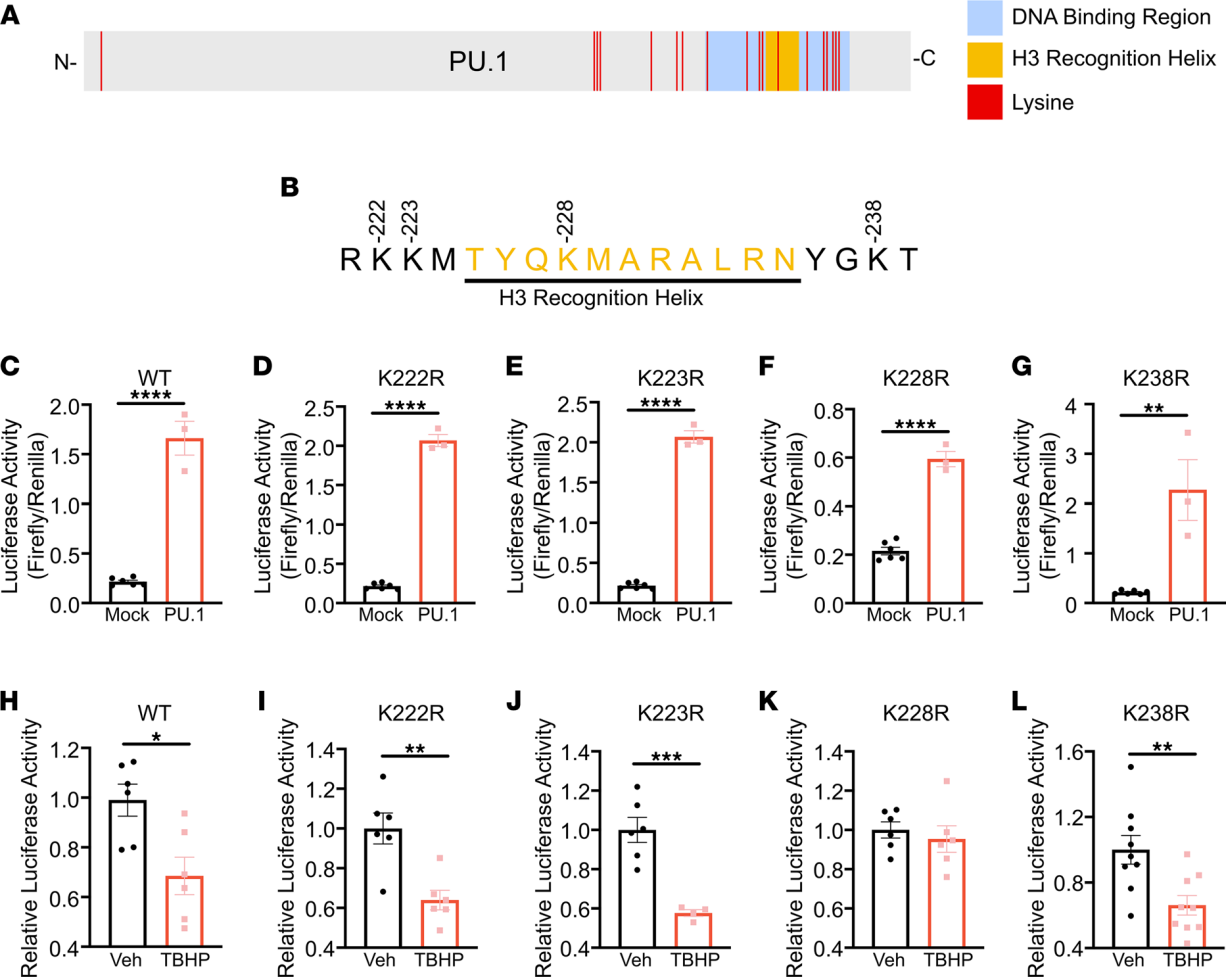
PU.1-K228R is insensitive to tBHP-mediated repression of C1qB. (**A**) Graphical representation of full-length PU.1 protein with the DNA binding region, H3 recognition helix, and individual lysines represented. (**B**) Mutated lysines within and flanking the H3 recognition helix are represented. HEK293T cells were cotransfected with C1qB273-Luciferase with empty expression vector (mock) or a PU.1 expression vector that expresses (**C**) wild-type (WT), (**D**) K222R, (**E**) K223R, (**F**) K228R, and (**G**) K238R. To determine the sensitivity of individual mutants to tBHP, HEK293T cells were cotransfected with C1qB273-Luciferase and a PU.1 expression vector and treated with vehicle or tBHP. Cells were cotransfected with (**H**) WT, (**I**) K222R, (**J**) K223R, (**K**) K228R, and (**L**) K238R. SV40-Renilla-Luciferase construct was used as an expression control. Data were analyzed with 2-tailed Student’s *t* test (*n* = 3–9, *P < 0.05, **P < 0.01, ***P < 0.001).

The DNA binding region of PU.1 contains 11 lysines (Figure 12A) (28). Within the H3 recognition helix, only a single lysine, K228, is present (Figure 12B). We mutated K228, in addition to the flanking lysines K223, K224, and K238, to arginine. Each mutant efficiently transactivated the C1qB273 promoter element (Figure 12, C–G), and like the wild-type PU.1, the K223, K224, and K238 mutants were sensitive to tBHP-mediated reduction of C1qB273-luciferase activation. In contrast, the K228R mutant was completely insensitive to transcriptional repression by tBHP (Figure 12, H–L).

### Monocyte-derived DCs are enriched in SLE and exhibit a reduction in population size and inflammatory gene programs with 2-HOBA treatment.

**Figure 13:**
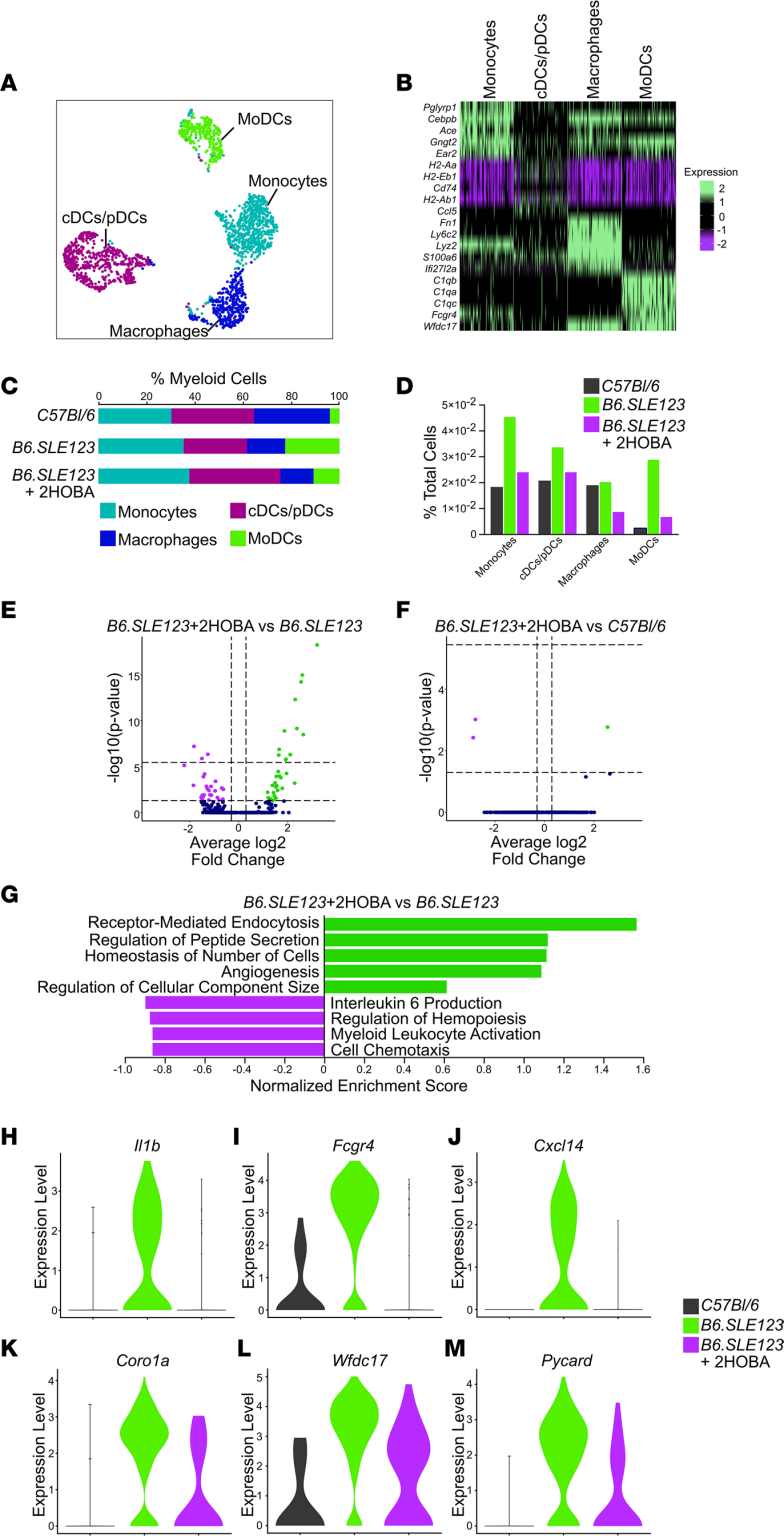
MoDCs exhibit a reduction in proinflammatory gene expression with 2-HOBA treatment. (**A**) Subclustering of myeloid cells with moDCs, cDC and cDCs/pDCs, monocytes, and macrophages represented. (**B**) Heatmap representing the top 5 markers for individual clusters. (**C**) Myeloid clusters represented as a percentage of total myeloid cells. (**D**) Myeloid clusters represented as percentage of total sequenced cells. (**E**) Volcano plot identifying differentially expressed genes in the moDC cluster between *B6.SLE123*+2-HOBA and *B6.SLE123* mice. (**F**) Volcano plot identifying differentially expressed genes in the moDC cluster of *B6.SLE123*+2-HOBA and *C57BL/6* control mice. (**G**) Gene ontology enrichment analysis of biological processes in the moDC cluster between *B6.SLE12*3+2-HOBA and *B6.SLE123*. Violin plots representing the expression of proinflammatory genes (**H**) *Il1b*, (**I**) *Fcgr4*, (**J**) *Cxcl14*, (**K**) *Coro1a*, (**L**) *Wfdc17*, and (**M**) *Pycard* in the moDC cluster.

To determine the role of isoLG scavenging on gene expression of individual antigen-presenting cell populations in SLE, we performed single-cell sequencing using the 5-prime 10x Genomics platform on splenocytes from *C57BL/6* mice and *B6.SLE123* mice treated with and without 2-HOBA. T cells were depleted prior to sequencing to enrich for myeloid populations. Twenty-eight clusters were identified (Supplemental Figure 9). Six myeloid clusters were identified based on the expression of the pan-myeloid marker *CST3*, whereas most populations were of the B cell lineage and were positive for *CD79a* (Supplemental Figure 10). Myeloid clusters were further analyzed for expression of the DC/monocyte markers *Itgax*, *Itgam*, *C1qa*, *Lyz2*, *Csf3r*, *Chil3*, *Il1b*, and *H2-Aa* and the neutrophil marker *S100a9* (Supplemental Figure 11). DC/monocyte clusters were analyzed independently. Four myeloid clusters were examined for expression of *C1qa*, *C1qb*, and *C1qc*; the monocyte marker *Csf1r*; macrophage markers *Lyz2*, *Itgam*, *Chil3*, and *Fcgr1*; the classical/plasmacytoid DC (cDC/pDC) marker *Itgax*; and the MHC-II isoform *H2-Aa* (Supplemental Figure 12). Individual clusters were functionally identified as monocytes, cDC/pDCs, macrophages, and monocyte-related DCs (moDCs) (Figure 13A). These clusters were identified by unique gene expression profiles (Figure 13B). Interestingly, moDCs uniquely expressed C1q subunits in addition to the DC genes *Itgax* and *H2-Aa*. These cells lacked traditional macrophage markers *Fcgr1*, which encodes CD68, and *Lyz2*, which encodes the lysozyme C-2 precursor protein. The monocyte lineage of this cluster was defined by the presence of *Csf1r*, which encodes the M-CSF receptor. Importantly, moDCs expanded in SLE as a percentage of both total myeloid cells and total sequenced cells when compared with *C57BL/6* control mice. Treatment with 2-HOBA restored this expansion to near control levels (Figure 13, C and D). Analysis of differentially expressed genes in moDCs between *B6.SLE123* mice with and without 2-HOBA treatment revealed 59 differentially regulated genes (Figure 13E). Only 3 differentially expressed genes were observed when comparing moDCs of *B6.SLE123* control mice versus *C57BL/6* mice treated with 2-HOBA (Figure 13F). No genes were significantly differentially expressed within the macrophage or cDC/pDC clusters (Supplemental Figure 13). Gene ontology analysis of significantly regulated genes within the moDC population revealed that 2-HOBA treatment attenuated inflammatory gene programs involved in IL-6 production, regulation of hemopoiesis, myeloid leukocyte activation, and cell chemotaxis. Gene ontology analysis of *B6.SLE123* mice treated with 2-HOBA revealed an increase in gene programs involved in receptor-mediated endocytosis, regulation of peptide secretion, homeostasis of number of cells, angiogenesis, and regulation of component size when compared with untreated *B6.SLE123* mice (Figure 13G). Examination of individual proinflammatory genes *Il1b*, *Fcgr4*, *Cxcl14*, *Coro1a*, *Wfdc17*, and *Pycard* within the moDC population revealed substantial upregulation in *B6.SLE123* mice with a reduction to or near control levels with 2-HOBA treatment (Figure 13, H–M).

### cDC/pDC populations exhibit no significant change in gene expression with 2-HOBA treatment.

**Figure 14:**
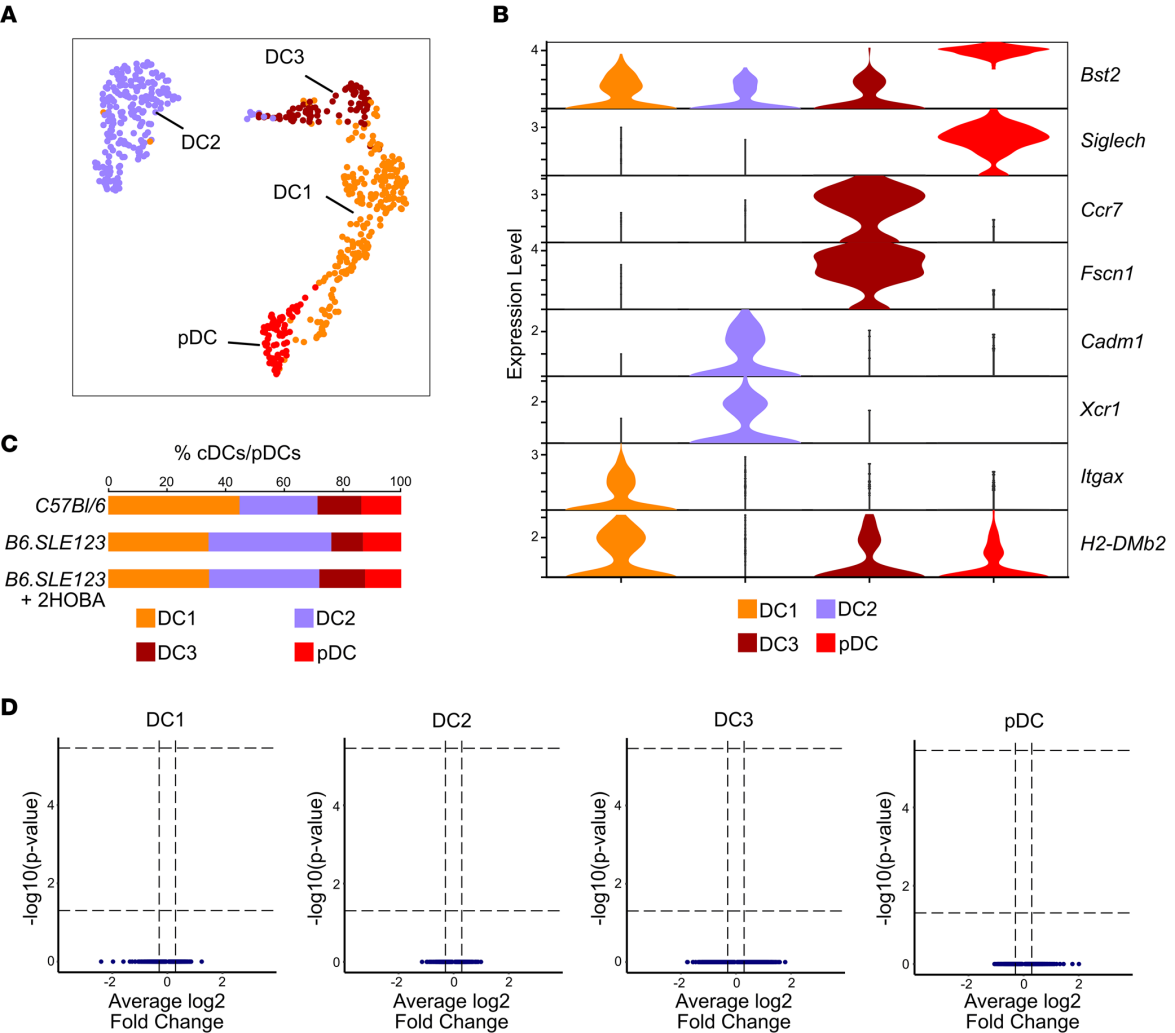
No significant differential gene regulation is identified in classical and pDCs in *B6.SLE123*+2-HOBA versus *B6.SLE123* mice. DCs were subclustered into DC1, DC2, DC3, and pDC. (**A**) Uniform manifold approximation and projection of DC clusters. (**B**) Violin cluster of markers for individual DC clusters. (**C**) DC clusters represented as a percentage of total cDCs/pDCs. (**D**) Volcano plots identifying differentially expressed genes in the DC clusters between *B6.SLE123*+2-HOBA and *B6.SLE123* mice.

We subclustered the cDC/pDC population into 4 clusters, DC1, DC2, DC3, and pDC, based upon previously described populations (Figure 14A) (29). Populations were evaluated for the DC1 markers *H2-Dmb2* and *Itgax*, DC2 markers *Xcr1* and *Cadm1*, DC3 markers *Fscn1* and *Ccr7*, and pDC markers *Siglech* and *Bst2* (Figure 14B). We observed no significant changes in cDC/pDC populations in *B6.SLE123* mice treated with 2-HOBA when compared to untreated *B6.SLE123* mice (Figure 14C). Differential gene expression analysis of *B6.SLE123* mice with and without 2-HOBA treatment revealed no significant changes in gene expression within any cDC/pDC subcluster (Figure 14D).

## Discussion

**Figure 15:**
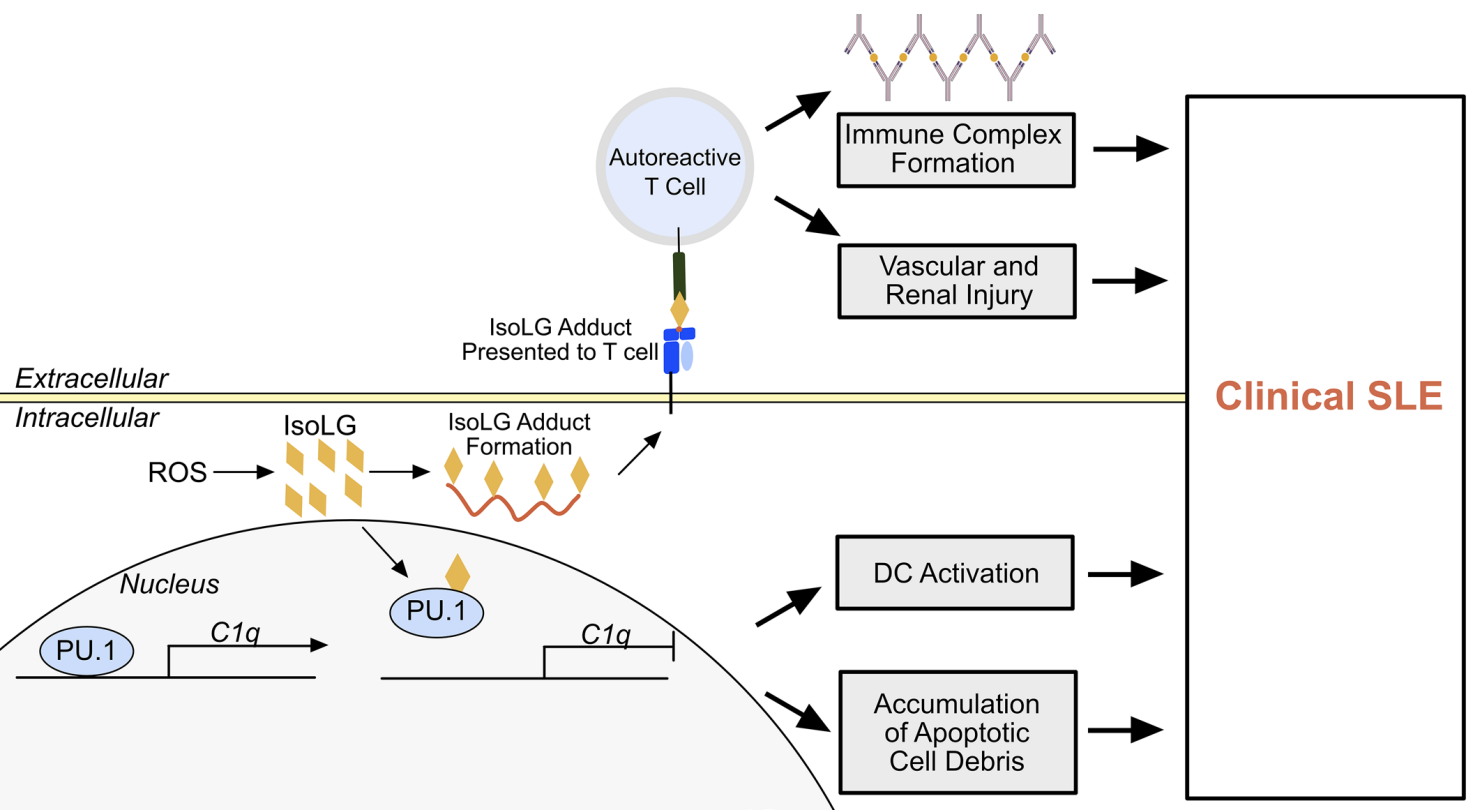
Paradigm of isoLG-induced immune activation in the development of SLE. Within an antigen-presenting cell, intracellular ROS results in formation of reactive isoLGs (orange diamonds), which adduct to protein. Intracellular proteins are processed and presented on the cell surface to autoreactive T cells, which then migrate to peripheral tissues and directly injure peripheral tissues and contribute to the development of immune complexes. Within the nucleus, isoLG adducts to PU.1, resulting in C1q transcriptional repression. This results in DC activation and accumulation of extracellular apoptotic debris. This process leads to the manifestation of clinical SLE.

SLE is a devastating disease that has pleiotropic clinical manifestations. Heretofore, the etiology of SLE has remained obscure. In the present study, we provide evidence that the accumulation of isoLGs within antigen-presenting cells has at least 2 important effects that promote SLE. The first is formation of autoantibodies against isoLG-adducted self-proteins, and the second is impairment of PU.1 binding to the C1q promoter, leading to reduction of C1q expression (Figure 15). Scavenging isoLGs markedly improved numerous aspects of SLE in 2 experimental animal models, including the formation of anti-dsDNA, renal dysfunction, parameters of renal inflammation, and hypertension. Thus, these findings provide insight into an etiology for SLE and suggest a specific therapy that may benefit this disease.

In prior studies, we and others have shown that adduction of proteins by isoLGs causes them to gain many features of neoantigens (11, 30). IsoLG-adducted proteins are presented in MHC-I complexes of DCs in hypertensive mice and mice with heart failure (11, 30). Adoptive transfer of DCs from hypertensive mice primes hypertension in recipient mice, and this is prevented if the donor mice are treated with 2-HOBA. Likewise, DCs from hypertensive mice promote proliferation of T cells, and this is also prevented if the donor mouse is treated with 2-HOBA (11, 20). Loading DCs with isoLG-modified proteins causes them to stimulate proliferation of memory T cells from hypertensive mice, a property not shared by other lipid oxidation products. Adoptive transfer of DCs in which isoLG formation has been stimulated primes hypertension in recipient mice. Our finding that both SLE-prone mouse strains *B6.SLE123* and *NZBWF1* and humans with SLE form IgG antibodies against isoLG-adducted proteins further supports the concept that these adducts act as antigens in SLE. This is further supported by the accumulation of isoLG adducts within antigen-experienced B cells.

In accord with the marked increase in isoLG adducts, we also observed an 8-fold increase in monocyte superoxide levels in humans with SLE. While superoxide can directly peroxidize lipids (31), other ROS derived from superoxide are likely to play a role, including peroxynitrite (32), singlet oxygen, hydrogen peroxide, and products of hydrogen peroxide (33). Thus, our findings should not be taken to indicate that superoxide is the only radical involved in formation of isoLGs in SLE but are compatible with the concept that superoxide or superoxide-derived ROS are involved. In keeping with this, oxidative injury has previously been implicated in the pathogenesis of SLE (5, 34, 35), and treatment with the superoxide scavenger tempol and the NADPH oxidase inhibitor apocynin attenuates hypertension in *NZBWF1* mice (9). This therapy did not, however, alter disease progression as measured by anti-dsDNA antibody titers. It is therefore possible that direct scavenging of isoLGs is more beneficial than nonspecific reduction of superoxide levels.

Future studies to examine the role of ROS in the pathogenesis of SLE are warranted. Although one obviously cannot infuse a toxic oxidant like tBHP in mice, animal models with selective deletion of antioxidant enzymes or overexpression of ROS-generating enzymes in antigen-presenting cells would be informative.

The reaction of 2-HOBA with isoLGs involves pyrrole formation between the dicarbonyl and its primary amine. We have previously shown that 2-HOBA does not scavenge superoxide or peroxynitrite and exhibits a slow rate of reactivity with malondialdehyde (11). Moreover, our prior studies indicate that 2-HOBA does not exhibit nonspecific immunosuppressant or antiinflammatory properties. 2-HOBA does not disrupt presentation of the ovalbumin OVA257-264 (SIINFEKL) peptide in murine DCs, suggesting that 2-HOBA treatment does not result in DC dysfunction (11). This agent has recently proved to be safe when given to healthy volunteers and had no effect on cyclooxygenase activity in them (36). Other isoLG-scavenging agents have also been developed that might prove effective in SLE.

Low complement levels have been used to gauge disease activity and are included in the SLEDAI (37). Given the strong association of C1q deficiency with the development of SLE, it is possible that repression of C1q gene expression by PU.1 isoLG adduction affects disease progression (12). Monocyte and DC-specific expression of C1q allows for efferocytosis, the process of removal of apoptotic cellular debris, which, when disrupted, results in exposure of immunogenic apoptotic cellular and nuclear bodies. Moreover, in the absence of ligation to extracellular material, C1q plays an autocrine role inducing a state of DC tolerance (14). C1q is consumed as a component of immune complexes and accumulates within renal structures in SLE. This consumption of C1q may result in a functional deficiency of C1q and result in immune activation. Additionally, given that patients with loss-of-function mutations in C1q subunits develop SLE, regulation of C1q expression and C1q production are also clearly important for the pathogenesis of SLE. Importantly, all 3 C1q subunits are controlled by a common promoter, and the activity of this promoter is highly regulated by the transcription factor PU.1 (15). Our finding that isoLG adduction to PU.1 represses its DNA binding and ability to transactivate provides a potentially novel mechanism underlying the genesis of SLE. PU.1 contains numerous lysine residues within the winged-helix-loop-helix DNA binding motif (28). Importantly, our data indicate that mutation of K228 uniquely eliminates PU.1 sensitivity to isoLG formation and that isoLG adduction of this site reduces PU.1-DNA interaction. Moreover, the role of isoLGs in suppression of the PU.1 target gene ZFP521 suggests the potential role of isoLGs as modulators of transcription in various disease states, including hematopoietic malignancies. Finally, given the marked accumulation of isoLGs within DCs from animal models of essential hypertension, it is possible that this mechanism also contributes to this disease process. Future studies to examine the role of PU.1 isoLG adduction in diverse pathological processes are warranted.

PU.1 has wide-ranging functions in hematopoiesis and plays an important role in myeloid and lymphoid cell fate decisions (38, 39). Yashiro et al. describe a role of PU.1 in the transcription of *itgax*, which encodes CD11c, a DC-specific marker, suggesting that PU.1 functions to promote DC differentiation (40). Conversely, *C1qB*, another PU.1 target gene, disrupts DC differentiation in an ex vivo differentiation system (15, 41). These observations suggest a feedback mechanism regulating DC differentiation and activation. We found an expansion of CD11c^+^ cells in SLE-prone mice that is abrogated with the isoLG scavenger 2-HOBA. Taken together, these data suggest that isoLG-mediated PU.1 inhibition occurs in terminally differentiated DCs. This results in suppression of C1q production leading to activation of differentiated DCs and augmentation of DC differentiation. This mechanism is further supported by the discovery that moDCs expand in SLE and exhibit a reduction in proinflammatory gene expression programs when exposed to 2-HOBA. Monocytes and moDCs have previously been shown to be important drivers of hypertension (42). Specifically, deletion of LysM-positive monocytes reduces aortic inflammation, vascular dysfunction, and oxidative stress in response to Ang II (43). Moreover, numerous cell types, including monocytes, have been shown to drive inflammation in lupus nephritis (44, 45). Importantly, the moDC population is by far the most responsive cell to 2-HOBA that we have examined. This further suggests that their activation is regulated by C1q levels and that they play an essential role in immune activation in SLE.

The specificity of 2-HOBA treatment for moDCs is remarkable. The lack of significant gene expression changes in other myeloid populations examined between SLE-prone mice with or without 2-HOBA treatment is strong evidence that 2-HOBA is specific and not a broad immunosuppressant. In fact, the lack of significant gene expression changes between control *C57BL/6* mice and *B6.SLE123* mice treated with 2-HOBA suggests that scavenging of isoLGs restores cell populations to a baseline level of gene expression. Downregulation of *Il1b* and genes involved in IL-6 production suggests a role of these cytokines in SLE pathogenesis. IL-6 specifically has been suggested to augment autoantibody production and T cell proliferation in the *NZBWF1* model of SLE and is enriched in plasma of patients with SLE (46–48).

SLE has protean clinical manifestations, laboratory abnormalities, and variable prognoses. The organ systems affected by SLE vary substantially, and it is therefore likely that human SLE has numerous contributing etiologies. We observed substantial heterogeneity in the level of isoLG adducts in monocytes of humans with SLE. It is therefore probable that isoLG adduct formation does not contribute to SLE in all patients. In preliminary analysis, we found that isoLG adducts inversely correlate with age. It is known that SLE is more severe in younger patients, and therefore isoLG adduct formation may reflect a more aggressive disease in this subgroup. Analysis of isoLG levels in humans might identify subgroups that would benefit from treatment with isoLG scavengers. Importantly, we found that isoLG adducts accumulate in antigen-presenting cells of *B6.SLE123* mice well before the onset of overt disease, and therefore measuring them in patients may aid in the diagnosis and allow for early intervention to prevent development of more advanced clinical manifestations.

The degree of IgG reactivity with isoLG adducts in humans with SLE correlates with the SLEDAI. It is likely that the production of these antibodies is driven by the accumulation and presentation of isoLG adducts within antigen-presenting cells. The presence of anti-isoLG adduct antibodies may be related to the degree of isoLG accumulation or the abduction of unique antigenic peptides that lead to immune activation. In any case, this discovery defines the presence of anti-isoLG antibodies as a surrogate marker for disease activity and suggests the use of an anti-isoLG adduct antibody ELISA as a method of SLE prognostication.

The possible predictive value of isoLG adducts in antigen-presenting cells of humans with SLE for developing hypertension requires additional study. We found in 2 murine models of SLE that isoLG adducts were increased in antigen-presenting cells before the onset of overt disease and scavenging of them beginning at this early time prevented the development of hypertension. We also observed a striking increase in isoLG adducts in monocytes of relatively young humans with SLE, who exhibited a low incidence of hypertension (Supplemental Table 1), and no correlation between isoLG adducts and blood pressure (Supplemental Figure 1). Most of the patients with SLE we studied were on angiotensin-converting enzyme inhibitors to prevent progression of renal disease. Angiotensin blockade has been shown to prevent the development of hypertension in non-SLE participants (49), and thus, could have masked hypertension in our cohort. Future studies to examine the efficacy of isoLG scavenging in preventing the development of hypertension as well as modifying other aspects of SLE are warranted.

In summary, in the present study, we have demonstrated that isoLGs play at least 2 roles in the genesis of SLE. The importance of isoLGs in the induction of essential hypertension and the associated renal inflammation suggests a shared pathway of autoimmunity between these conditions. While the role of isoLGs in other autoimmune conditions has yet to be studied, scavenging of isoLGs with molecules such as 2-HOBA may also be useful in these conditions.

## Methods

### Animals studied.

*C57BL/6J*, *NZW/LacJ*, *B6.NZMSLE1/SLE2/SLE3*, and *NZBWF1/J* were obtained from The Jackson Laboratory. Female mice were used for all experiments. Acetic acid salt of 2-HOBA was synthesized as reported previously (50). Animals were treated with 2-HOBA (1 g/L of drinking water) beginning at 7 weeks of age. 2-HOBA was thawed from a frozen stock 3 times per week, at which time animal water was changed and was shielded from light in amber water bottles. Radiotelemeters were implanted into animals at 30 weeks of age. Blood pressure was monitored noninvasively using tail cuffs and invasively using radiotelemetry as previously described (11, 51). The 4-hour urinary excretion assay was performed using metabolic cages at 30 weeks of age as previously described (19). Animals were weighed and injected with saline at a volume (mL) of 10% of body weight (mg). Urinary volume was measured following 4 hours in the absence of supplemented water. Animals were sacrificed at 32 weeks of age. Urinary albumin and creatinine were measured using commercially available test kits from Exocell. Urinary NGAL was measured using a commercially available ELISA (Abcam). Plasma BUN was measured using a commercially available kit (Invitrogen). dsDNA and total IgG were measured using commercially available ELISAs (Alpha Diagnostic). Seven-week-old animals were sacrificed for studies of animals without overt SLE and for RNA sequencing. For RT-PCR, animals were treated with 2-HOBA beginning at 7 weeks old for a total of 6 weeks and sacrificed at 13 weeks of age.

### Histology.

Kidneys were fixed in 10% buffered formalin. A Jones’ sliver stain or PAS stain was performed by the Vanderbilt Translational Pathology Shared Resource. Slides were scanned and evaluated in a blinded analysis. A total of 30 nephrons were scored per kidney for capillary hypercellularity by counting capillary nuclei based on the semiquantitative scale of 0 (normal, 0–5 nuclei), 1 (mild, 5–15 nuclei), and 2 (severe, >15 nuclei). Glomeruli were also scored for the presence of immune complex deposition based on the presence of spikes/holes in the subepithelium on Jones’ silver stain by light microscopy as previously described (52). Tubules were scored by evaluating 10 fields per animal at 40× original magnification on slides stained with PAS. Each field was scored for dilated tubules, loss of proximal tubule brush border, cellular vacuolization, tubular degeneration, and casts. Each field was scored 0 (normal, no abnormalities observed), 1 (≤25% abnormal field), 2 (≤50% abnormal), 3 (≤75% abnormal), or 4 (100% abnormal). All scores were summed and divided by 10 to generate a tubular injury score for each animal.

### Flow cytometry.

For human samples, a single-cell suspension of PBMCs was prepared and analyzed as previously described (11, 53, 54). PBMCs were harvested by Ficoll gradient separation as previously described (55). Live/dead cell staining was performed with 7-aminoactinomycin D. Cells were then stained with the following surface antibodies: PE anti-CD45 (Thermo Fisher Scientific catalog 12-0451-82), APC/Cy7 anti-CD14 (BD Biosciences 561709), PE/Cy7 anti-CD11c (Thermo Fisher Scientific 25-0116-42), APC anti-CD83 (Thermo Fisher Scientific BDB551073), and Brilliant Violet 510 anti-CD86 (BD Biosciences 563460).

Tissue homogenates from mice were filtered through a 40 μm filter (BD Biosciences). Single-cell suspensions were stained for flow cytometry and run in 3 panels using the following antibodies and fluorophores. Pacific LIVE/DEAD Fixable Violet Dead Cell Stain (Thermo Fisher Scientific) was used for the lymphoid panel. T cell panel utilized the following surface antibodies: FITC anti-CD45 clone 30-F11 (BioLegend), PE-Cy7 anti-CD3 clone 17A2 (BioLegend), APC anti-CD4 clone RM4-5 (BD Biosciences), and Amcyan anti-CD8 clone 53-6.7 (BioLegend). B cell panel utilized the following surface antibodies PE-Cy7 anti-CD45 clone 30-F11 (BioLegend), APC anti-CD19 clone 1D3/CD19 (BioLegend), PE anti-CD44 clone IM7 (BioLegend), and Amcyan anti-CD138 clone 281-2 (BD Biosciences). Myeloid panel utilized the Zombie NIR Fixable Viability Kit (BioLegend) and the following surface antibodies: Pacific Orange anti-CD45 clone 30-F11 (Thermo Fisher Scientific), BV510 F4/80 anti-F4/80 clone T45-2342 (BD Biosciences), APC anti-MerTK clone 108928 (R&D Systems), AF700 anti-CD11b clone M1/70 (BioLegend), PE/Dazzle 594 anti-CD64 clone X54-5/7.1 (BioLegend), PE anti-Lyc6c clone HK1.4 (BioLegend), PE/Cy7 anti-IAb clone AF6-120.1 (BioLegend), and APC/Cy7 anti-CD11c clone N418 (BioLegend). A known quantity of calibration (counting) beads was added to each sample before analysis. Samples were run on a BD FACSCanto II system or a Cytek Aurora system and analyzed using FlowJo software. Gates were set using fluorescence minus one controls. Results were normalized using the bead count and expressed as the number of cells per organ. For isoLG adduct–positive cells and plasma cells, cells are represented as total percentage of parent population.

For intracellular staining, cells were fixed and permeabilized using a commercially available cell permeabilization kit (Thermo Fisher Scientific). Intracellular staining for the B cell panel used peridinin chlorophyll protein anti-Ig κ light chain clone 187.1 (BD Biosciences). Intracellular staining for isoLG adducts was performed using the single-chain antibody D11 (11), which recognized isoLG lysines independent of adjacent amino acids. The D11 antibody was labeled with a fluorochrome using the APEX Alexa Fluor 488 Antibody Labeling Kit (Thermo Fisher Scientific). All antibodies and fluorophores employed are summarized in Supplemental Table 6.

### Anti-IsoLG adduct antibody ELISA.

For mice, whole kidney protein was prepared in nondenaturing lysis buffer (20 mM Tris pH 8.0, 137 mM NaCl, 1.0% NP-40, 2 mM EDTA). Unreacted isoLG was synthesized as previously described (56). A total of 100 μG of protein was incubated with isoLG at a concentration of 100 μM isoLG overnight at 4°C. Immulon 2 HB plates (Immunochemistry Technologies) were then coated with D11 at a concentration of 50 μg/mL in coating buffer (1.5 g Na2CO_3_, 2.93 g NaHCO_3_, to 1 L, pH 9.6) overnight at 4°C at a volume of 100 μL. Plates were then washed 3 times with wash buffer (PBS, 0.05% Tween) and then blocked with blocking buffer (PBS + 3% BSA, 3 mM EDTA, and 0.1% gelatin) at 37°C for 1 hour. Wells were then incubated with 50 μg/mL isoLG-adducted protein in binding buffer (PBS + 2% BSA, 3 mM EDTA, 0.05% Tween 20, 0.1% gelatin) for 1 hour at 37°C at a volume of 100 μL. Plates were then washed 4 times with wash buffer. Serum was diluted 1:100 in protein binding buffer and then incubated on wells for 1 hour at 37°C at a volume of 100 μL. Plates were washed 4 times with wash buffer and incubated with Protein-G–HRP conjugate (Thermo Fisher Scientific) at a dilution of 1:100 in secondary antibody diluent (PBS + 1% BSA, 0.05% Tween 20) for 1 hour at 37°C. Plates were washed 3 times and 100 μL of TMB substrate solution (Thermo Fisher Scientific) was added for detection. Plates were incubated at room temperature in the dark for 30 minutes. Stop solution (Thermo Fisher Scientific) was added, and absorbance was measured at 450 nM. For human plasma samples, PBMC protein was prepared from a healthy woman in nondenaturing lysis buffer and adducted with isoLG as described above. Plasma was diluted 1:10,000 in binding buffer prior to incubation. The remainder of the protocol is described above.

### Studies of isoLG adducts in humans.

Participants with SLE and controls were recruited from individuals enrolled in 3 larger studies. Participants were 18–56 years of age, met classification criteria for SLE as determined by a rheumatologist, and provided written informed consent (57). Exclusion criteria included concomitant diabetes mellitus, recent change in medication, regular daily use of opioids/alcohol/other drugs of abuse, severe psychiatric disease, refusal to give informed consent, or pregnancy. Control participants were recruited by advertisement to the Vanderbilt Clinical Trials Center as part of the Immune Mechanisms of Essential and Lupus-Related Hypertension study. These control participants were 29–64 years old. Exclusion criteria included confirmed or suspected renal, renovascular, or endocrine causes of secondary hypertension; concomitant diabetes; concomitant illness requiring corticosteroids or immunosuppressants; recent (within 3 months) vaccination against any infectious agent; active ongoing malignancy; severe psychiatric disorders; or HIV/AIDS. All participants gave written informed consent.

### Mass spectrometry for isoLG-lysine and superoxide in monocytes.

IsoLG-lysine adducts were detected in human monocytes as previously described (58). Superoxide was quantified by monitoring the conversion of dihydroethidium to 2-hydroxyethidium using HPLC as previously described (59).

### EMSA.

dsDNA probes with 3′ biotin labels were purchased from IDT. The sense strand sequences for utilized probes are PU.1 5′ CCCGCCTCTGGGGAAGGGAACTTCCGCT 3′ and IRF8 5′ TGGGTTGCAGAAATAGGACCTGAAACTGCCTGAGG 3′. A total of 10 μg of recombinant human PU.1 (Abcam) and IRF8 (OriGene) were incubated with isoLG at a final concentration of 100 μM for 1 hour at room temperature, after which 2-HOBA was added for a final concentration of 5 mM to quench excess isoLG. 2-HOBA was also added to unadducted protein as a control. dsDNA probes were annealed using a thermocycler. Binding reactions were performed using the LightShift chemiluminescence EMSA kit following the manufacturer’s instructions (Thermo Fisher Scientific). Briefly, 80 nmol of annealed probe was incubated with 1 μg of PU.1 or IRF8 in the presence of poly(dI-dC), to abrogate nonspecific binding. Samples were incubated for 20 minutes at room temperature and separated on a 5% polyacrylamide 0.5× Tris-borate-EDTA buffer gel. The gel was transferred to a nylon membrane and imaged according to the manufacturer’s instructions.

### ChIP and immunoblot.

Healthy controls were recruited to the Clinical Trials Center at Vanderbilt University Medical Center. PBMCs were separated with a Ficoll gradient, and monocytes were isolated with positive selection utilizing commercially available CD14 magnetic microbeads (Miltenyi Biotec). Cells were resuspended to a density of 1 × 10^6^ monocytes/mL in medium containing vehicle, or 30 ng/mL of GM-CSF (BioLegend), and cultured for 24 hours. Following 24-hour treatment cells were resuspended in a solution containing vehicle, GM-CSF, or GM-CSF supplemented with 100 μM tBHP and incubated for 30 minutes. Following incubation, tBHP was removed, and cells were resuspended in medium supplemented with vehicle or GM-CSF (30 ng/mL) and incubated for an additional 24 hours. ChIP was then performed utilizing the Sigma Imprint Chromatin Immunoprecipitation Kit following the manufacturer’s instructions. ChIP was performed with the anti-PU.1 antibody obtained from Abcam, catalog ab76543. Endpoint PCR was performed with OneTaq Polymerase (New England Biolabs) utilizing the following primer set: 5′ GTCAGGGGAAAGCCCTT 3′ and 5′ CCGAAGTTCCCT 3′. Gel images were quantitated using ImageJ (NIH). PCR products were sequence confirmed by gel purification and DNA sequencing.

For immunoblot, cells treated with GM-CSF or cotreated with GM-CSF and tBHP as described above were harvested. Cells were lysed with RIPA buffer (Sigma), and 50 μg of protein was separated by SDS-PAGE. Gels were transferred to a nitrocellulose membrane and incubated with 1:1000 of the anti-PU.1 antibody obtained from Abcam, catalog ab76543. The blot was stripped with Restore Western Blot Stripping Buffer (Thermo Fisher Scientific) and reprobed with anti–β-actin–peroxidase antibody clone AC-15 (Sigma) at a 1:20,000 dilution. Imaging was performed with a BioRad gel imaging station.

### Cell culture, Et-2-HOBA treatment, and luciferase assays.

HEK293T cells were obtained from American Type Culture Collection. pGL3-Basic-ZPF521 and pCMVSport6-PU.1 were gifts from Kathryn Hentges (University of Manchester, Manchester, United Kingdom) (25). C1qB273 was subcloned into pGL3-Basic using a G-block purchased from IDT. pReceiver-M12-PU.1 and all mutations were purchased from Genecopoeia. pRL-SV40 Renilla luciferase expression vector was purchased from BioRad. Cells were cultured to 50%–70% confluence and transfected using Lipofectamine 2000 (Invitrogen) in 12-well tissue culture–treated dishes (Corning) following the manufacturer’s instructions. Empty pReceiver vector was used for mock transfections. Et-2-HOBA was synthesized from 4-ethylphenol as previously described (50). Twenty-four hours following transfection, cells were pretreated with Et-2-HOBA for 2 hours at a concentration of 200 μM. Fresh culture medium was used as vehicle. Cells were then treated for 30 minutes with tBHP at a concentration of 100 μM in the presence or absence of Et-2-HOBA. Medium was then replaced with fresh medium containing Et-2-HOBA or vehicle. Cells were incubated for an additional 24 hours and a luciferase assay was performed using the BioRad Dual-Luciferase Reporter Assay following the manufacturer’s instructions.

### RNA sequencing.

Seven-week-old female *B6.SLE123* and control *C57BL/6* were obtained from The Jackson Laboratory. Animals were euthanized and CD11c-positive splenocytes were isolated as previously described (60). RNA was prepared utilizing the RNeasy Micro Kit (QIAGEN) with an RNase-free DNase treatment following the manufacturer’s instructions. cDNA library construction and RNA sequencing were performed by Vanderbilt Technologies for Advanced Genomics, Vanderbilt University Medical Center, Nashville, Tennessee,USA. Library preparation and RNA sequencing were performed as previously described (61).

### RNA-sequencing data analysis.

Quality control was performed on all sequencing reads using FastQC package developed by the Babraham Institute bioinformatics group. Reads with poor quality were trimmed and adapter sequences were removed by cutadapt (62). Reads were then aligned to mouse genome (mm10) using STAR (63) and quantified by featureCounts (64). Alignment quality were checked by QC3 (65). Significantly differentially expressed genes with FDR-adjusted *P* < 0.05 and absolute fold change > 2.0 were detected by DESeq2 (66). Heatmap3 (67) was used for cluster analysis and visualization. Genome Ontology and KEGG pathway overrepresentation analysis was performed on differentially expressed genes using the WebGestalt R package (68).

### Real-time RT-PCR.

Total RNA was extracted using the RNeasy Micro Kit (QIAGEN) following the manufacturer’s instructions. The concentration of the isolated RNA was determined by UV spectrophotometry (DeNovix Spectrophotometer). Reverse transcription was performed using TaqMan Reverse Transcription reagents (Thermo Fisher Scientific). Real-time RT-PCR was performed as previously described (16). Gene expression values were calculated based on the comparative Ct normalized to the expression values of GAPDH mRNA and displayed as fold change normalized to control for each transcript.

### Single-cell sequencing.

Single-cell sequencing reported here was performed as part of a larger study. Seven-week-old female *B6.SLE123* mice were obtained from The Jackson Laboratory and treated with or without 2-HOBA for 6 weeks. Age-matched *C57BL/6* female mice were used as controls. Mice were sacrificed and spleens were harvested and processed into single-cell suspensions. T cells were depleted using anti-CD3 microbeads (Miltenyi Biotec). Cells from 3 mice per condition were combined, and single cells from each condition were hashed using TotalSeq-C antibodies (BioLegend). Single-cell sequencing was performed using the Chromium Single-Cell v2 5′ Chemistry Library, Gel Bead, Multiplex, and Chip Kits (10x Genomics) according to the manufacturer’s protocol. A total of 12,000 cells were targeted per well. Libraries were then sequenced utilizing the NovaSeq 6000 platform (Illumina). Raw base call files were demultiplexed and mapped using the Cell Ranger Single Cell Gene Expression v.6.0.0 software (10x Genomics). We used Seurat v4.0.0 in R v4.0.4 for data normalization, cell filtering, dimensionality reduction, clustering, and gene expression analysis using default parameters. Cells were included with RNA counts greater than 200 and less than 3500 and a mitochondrial content less than 15%. The R script is available upon reasonable request. Gene ontology analysis was performed with the WebGestalt tool kit (68).

### Data availability.

Results for bulk and single-cell RNA sequencing were uploaded to the National Center of Biotechnology Information Gene Expression Omnibus database (accession numbers GSE200485 and GSE200588, respectively).

### Statistics.

All data are expressed as mean ± SEM. Comparisons made between 2 variables were performed using 1- and 2-tailed Student’s *t* tests or Mann-Whitney *U* test depending on normality of distribution. Normality of distribution of data was confirmed using the D’Agostino-Pearson normality test. Comparisons among more than 2 variables were performed with 1-way ANOVA with Tukey’s post hoc test. To compare differences in blood pressure and C1q gene expression, 2-way ANOVA followed by Tukey’s post hoc test was used.

### Study approval.

All animal procedures were approved by Vanderbilt University Medical Center’s Institutional Animal Care and Use Committee, and the mice were housed and cared for in accordance with the *Guide for the Care and Use of Laboratory Animals*, US Department of Health and Human Services (National Academies Press, 2011). The institutional review board of Vanderbilt University Medical Center approved the human studies (IRB 150544, 180256, and 130979).

## Author contributions

DMP and DGH designed the study. DMP, NDLV, JK, JPVB, SSD, SD, AD, LX, AK, WC, and MA performed experiments and acquired the data. DMP, NDLV, DGH, SD, AD, WC, SZ, CDS, and SSD analyzed the data. DMP, MJO, CMS, LJC, and JMW obtained clinical samples and acquired clinical data. VA provided Et-2-HOBA and valuable expertise regarding isoLGs. ABF provided expertise on renal histology. DMP and DGH wrote the manuscript. All authors reviewed and revised the manuscript. DGH supervised the study.

## Supplementary Material

Supplemental data

## Version 1 05/24/2022

In-Press Preview

## Version 2 07/08/2022

Electronic publication

## Version 3 07/21/2022

Fig 12 was a duplicate of fig 2. It has been corrected.
