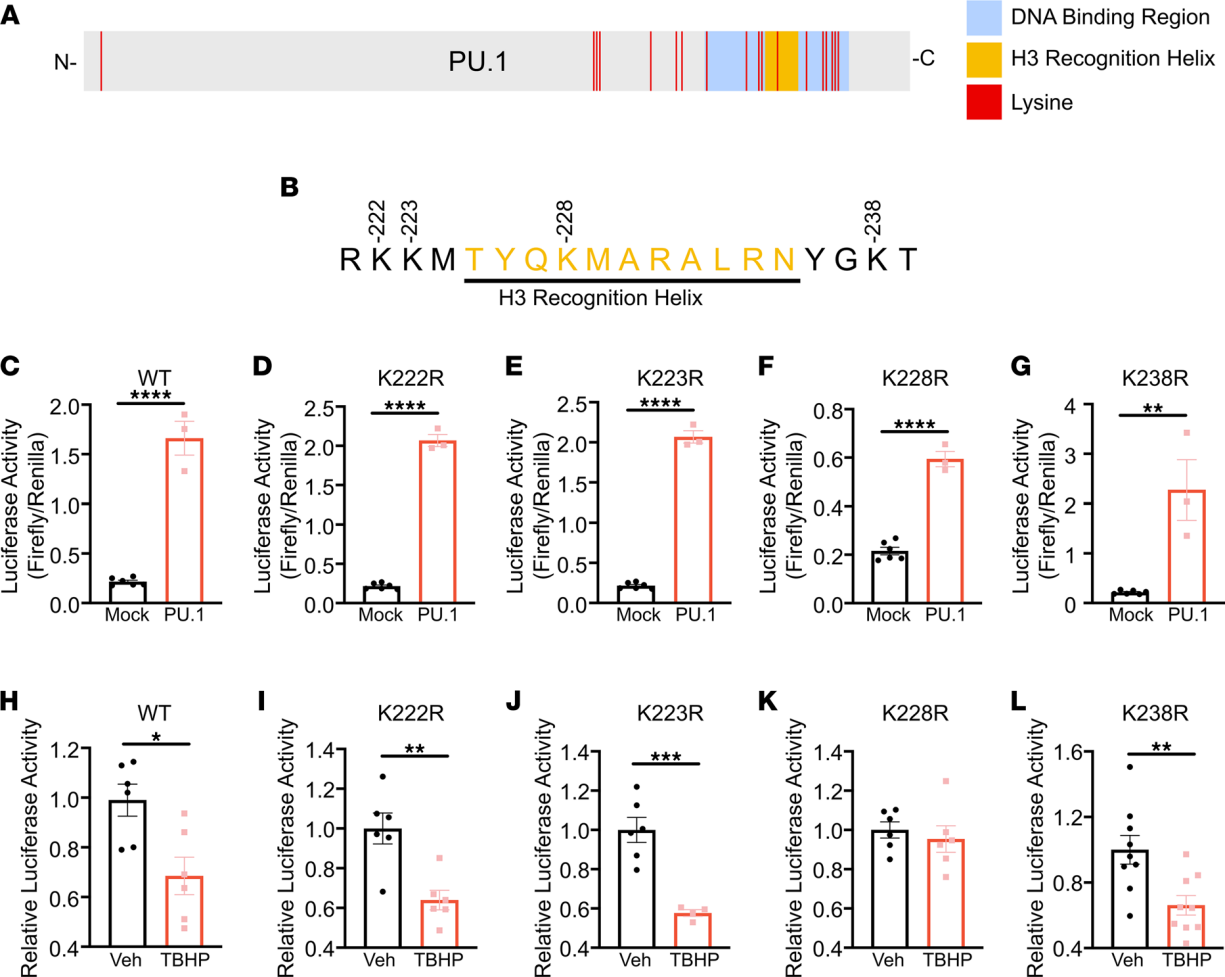# Isolevuglandins disrupt PU.1-mediated C1q expression and promote autoimmunity and hypertension in systemic lupus erythematosus

**DOI:** 10.1172/jci.insight.163757

**Published:** 2022-08-08

**Authors:** David M. Patrick, Néstor de la Visitación, Jaya Krishnan, Wei Chen, Michelle J. Ormseth, C. Michael Stein, Sean S. Davies, Venkataraman Amarnath, Leslie J. Crofford, Jonathan M. Williams, Shilin Zhao, Charles D. Smart, Sergey Dikalov, Anna Dikalova, Liang Xiao, Justin P. Van Beusecum, Mingfang Ao, Agnes B. Fogo, Annet Kirabo, David G. Harrison

Original citation: *JCI Insight*. 2022;7(13):e136678. https://doi.org/10.1172/jci.insight.136678

Citation for this erratum: *JCI Insight*. 2022;7(15):e163757. https://doi.org/10.1172/jci.insight.163757

During the preparation of this manuscript, Figure 12 was inadvertently replaced with an incorrect figure. The correct Figure 12 is below. The article has also been corrected online.

The *JCI* regrets the error.

## Figures and Tables

**Figure 12:**