# Supplementary material for: Isolevuglandins disrupt PU.1-mediated C1q expression and promote autoimmunity and hypertension in systemic lupus erythematosus
Source: JCI Insight. 2022 Jul 8;7(13):e136678. doi: 10.1172/jci.insight.136678 (PMC9310530; doi:10.1172/jci.insight.136678)
Supplement: Supplemental data [file jciinsight-7-136678-s245.pdf]

Supplemental Information

Isolevuglandins disrupt PU.1 mediated C1q expression and promote autoimmunity and hypertension in systemic lupus erythematosus

Authors: David M. Patrick[1,2,3], Néstor de la Visitación[2,4], Jaya Krishnan[2], Wei Chen[2], Michelle J. Ormseth[3,5], C. Michael Stein [2,5], Sean S. Davies[2], Venkataraman Amarnath[2], Leslie J. Crofford[5], Jonathan M. Williams[5], Shilin Zhao[6], Charles D. Smart[2,7], Sergey Dikalov[2], Anna Dikalova[2], Liang Xiao[2], Justin P. Van Beusecum[2,8,9], Mingfang Ao[2], Agnes B. Fogo[10], Annet Kirabo[2], David G. Harrison[2,3,*]

Affiliations: [1] Department of Veterans Affairs, Nashville, Tennessee. [2] Division of Clinical Pharmacology, Department of Medicine, Vanderbilt University Medical Center, Nashville, Tennessee, USA. [3] Division of Cardiovascular Medicine, Department of Medicine, Vanderbilt University Medical Center. [4]Department of Pharmacology, University of Granada, Granada, Spain. [5]Division of Rheumatology and Immunology, Department of Medicine, Vanderbilt University Medical Center. [6]Vanderbilt Center for Quantitative Sciences, Vanderbilt University Medical Center. [7]Department of Molecular Physiology and Biophysics. [8]Department of Veterans Affairs, Charleston South Carolina. [9]Division of Nephrology, Medical University of South Carolina, Charleston, South Carolina. [10]Department of Pathology, Microbiology and Immunology, Vanderbilt University Medical Center.

Conflicts of Interest: DMP and DGH have a patent pending for the use of isoLG scavengers to treat SLE.

***Address for correspondence:**
David G. Harrison, MD
220 Pierce Avenue
Room 536 Robinson Research Building
Vanderbilt University
Nashville, TN 37232-6602
Telephone: 615-322-3304/ Fax: 615-875-3297
e-mail: David.g.harrison@vumc.org

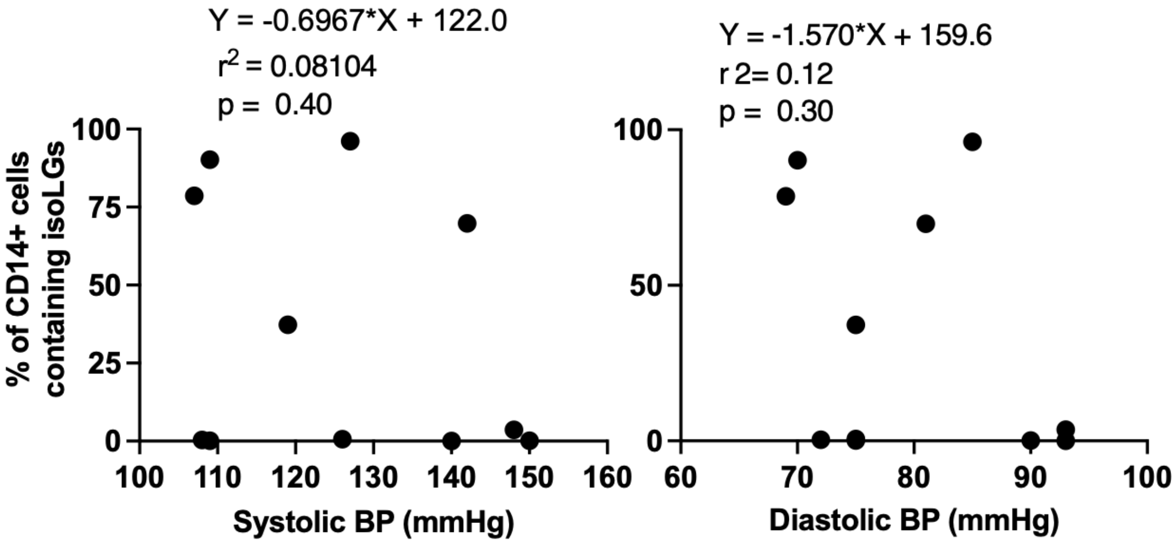

Supplemental Figure 1: Percent of CD14+ monocytes containing isoLG-adducts among subjects with SLE. Demographics of these subjects are presented in Supplemental Table 1.

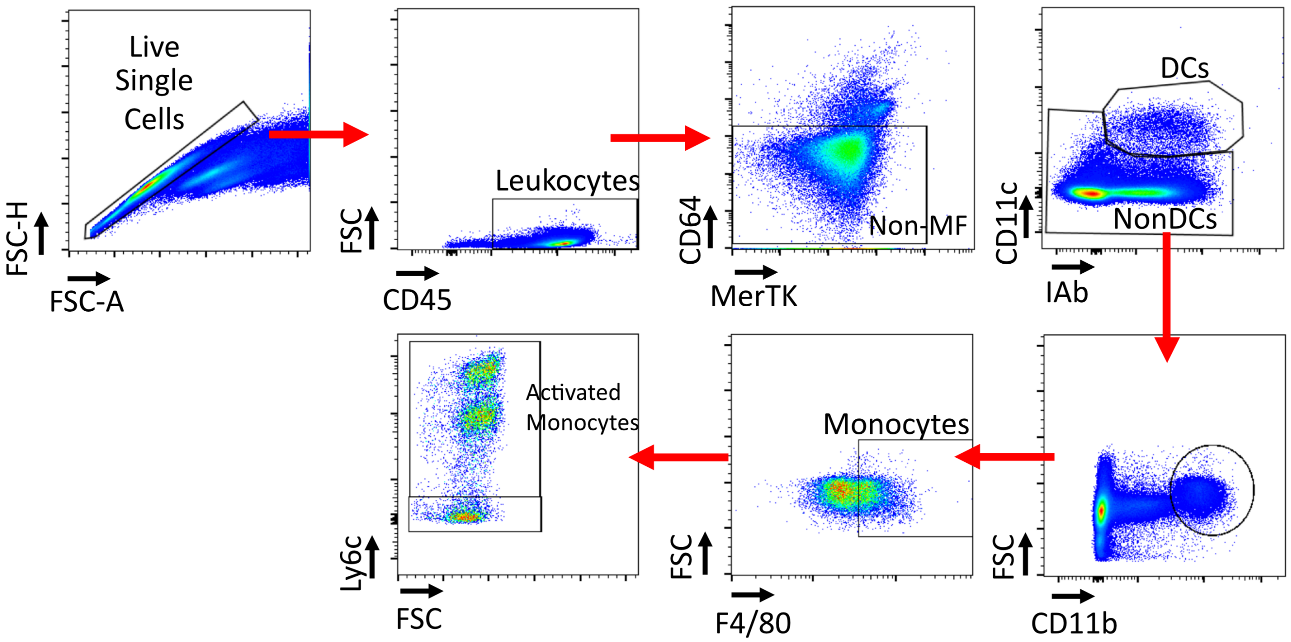

Supplemental Figure 2: Gating strategy for dendritic cells and monocytes.

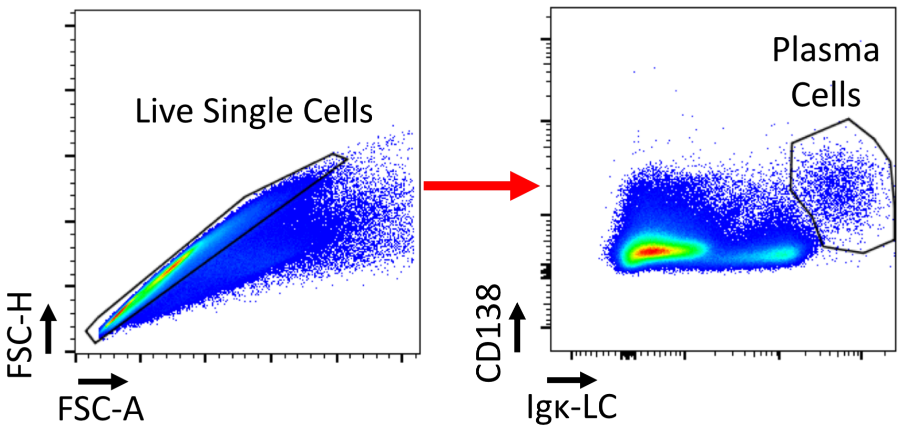

Supplemental Figure 3: Gating strategy for plasma cells.

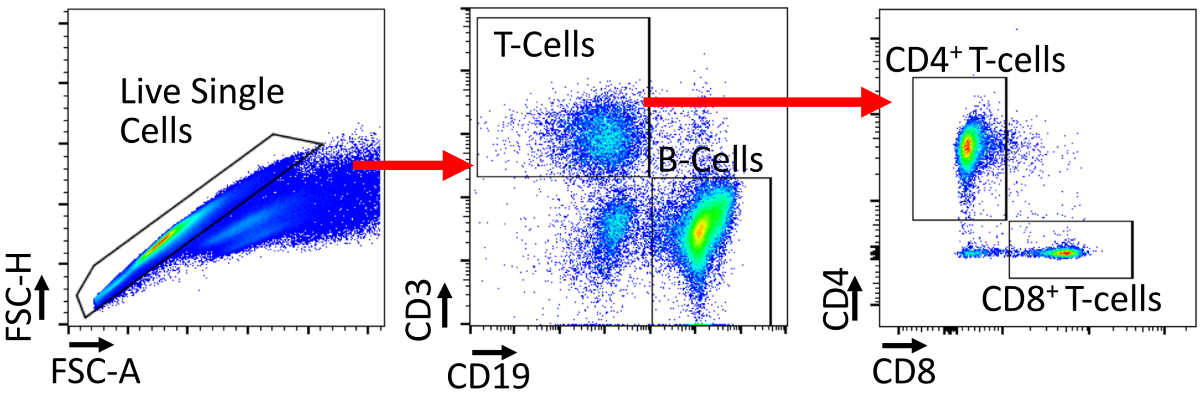

Supplemental Figure 4: Gating strategy for B-cells and T-cells.

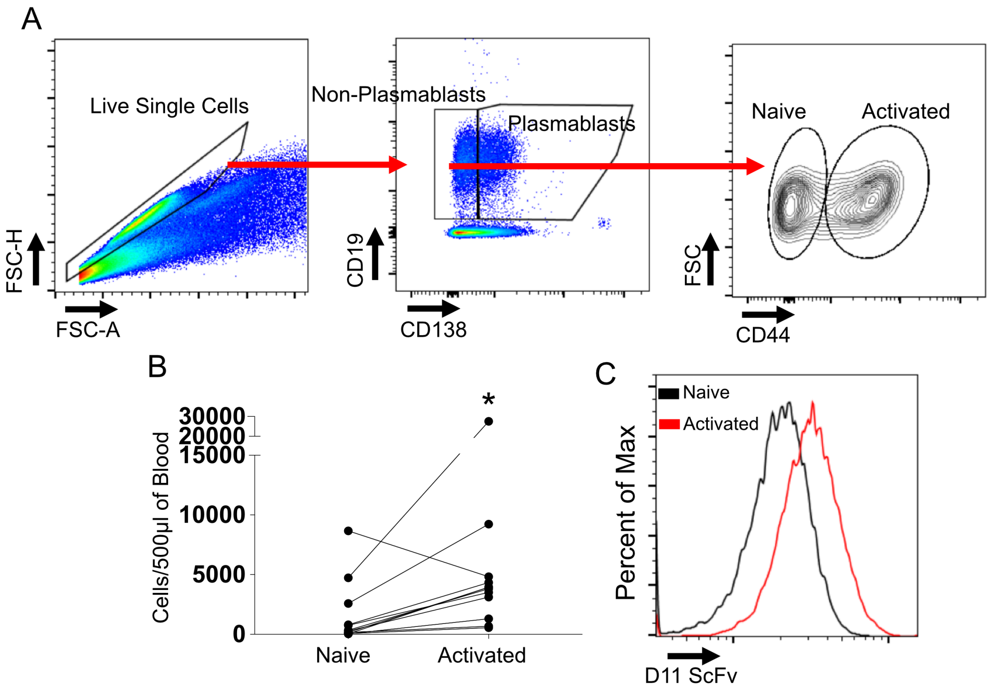

Supplemental Figure 5: IsoLG-adducts are enriched in peripheral activated B cells of SLE prone mice. Cells were isolated at the time of sacrifice from 32-week old *B6.SLE123* mice. Activated B cells (CD44^{hi}) in peripheral blood exhibit augmented isoLG adduct accumulation compared to naive B cells. **(A)** Gating strategy to define activated vs naive B cells. Gating was performed on non-plasmablast B cells. **(B)** IsoLG-adduct containing cells in peripheral blood of *B6.SLE123*. Data were analyzed with a paired T-test (n = 12, $^{*}P < 0.05$) **(C)** Representative histograms of isoLG adduct levels in activated vs naive B cells.

=

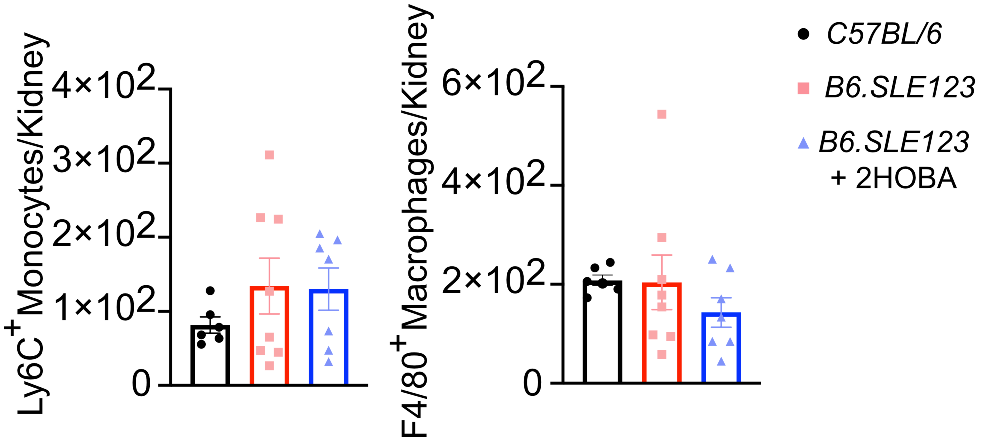

Supplemental Figure 6: Renal Infiltrating Ly6C$^+$ monocytes and F4/80$^+$ macrophages are unchanged by SLE or 2HOBA treatment. Cells were isolated at the time of sacrifice from 32-week old *B6.SLE123* mice and examined by flow cytometry. Data were analyzed using 1-way ANOVA (*n* = 6-8).

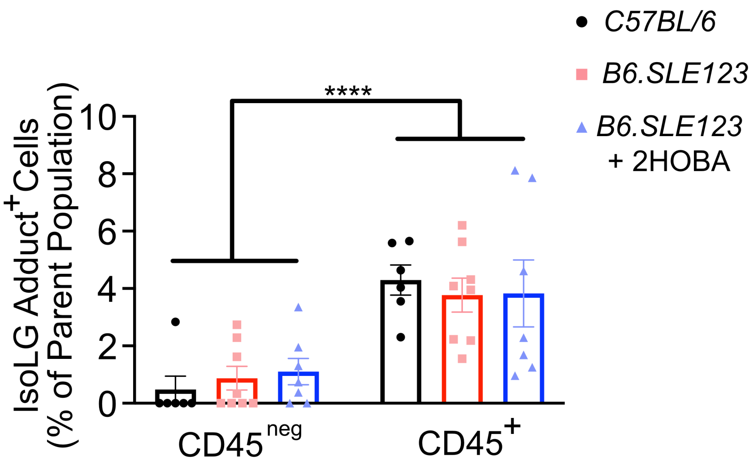

Supplemental Figure 7: Renal Infiltrating CD45^{+} cells exhibit augmented isoLG-adducts compared to CD45^{neg} cells. Cells were isolated at the time of sacrifice from 32-week old *B6.SLE123* mice and examined by flow cytometry. Data were analyzed using 2-way ANOVA (n = 6-8, **** $P < 0.0001$).

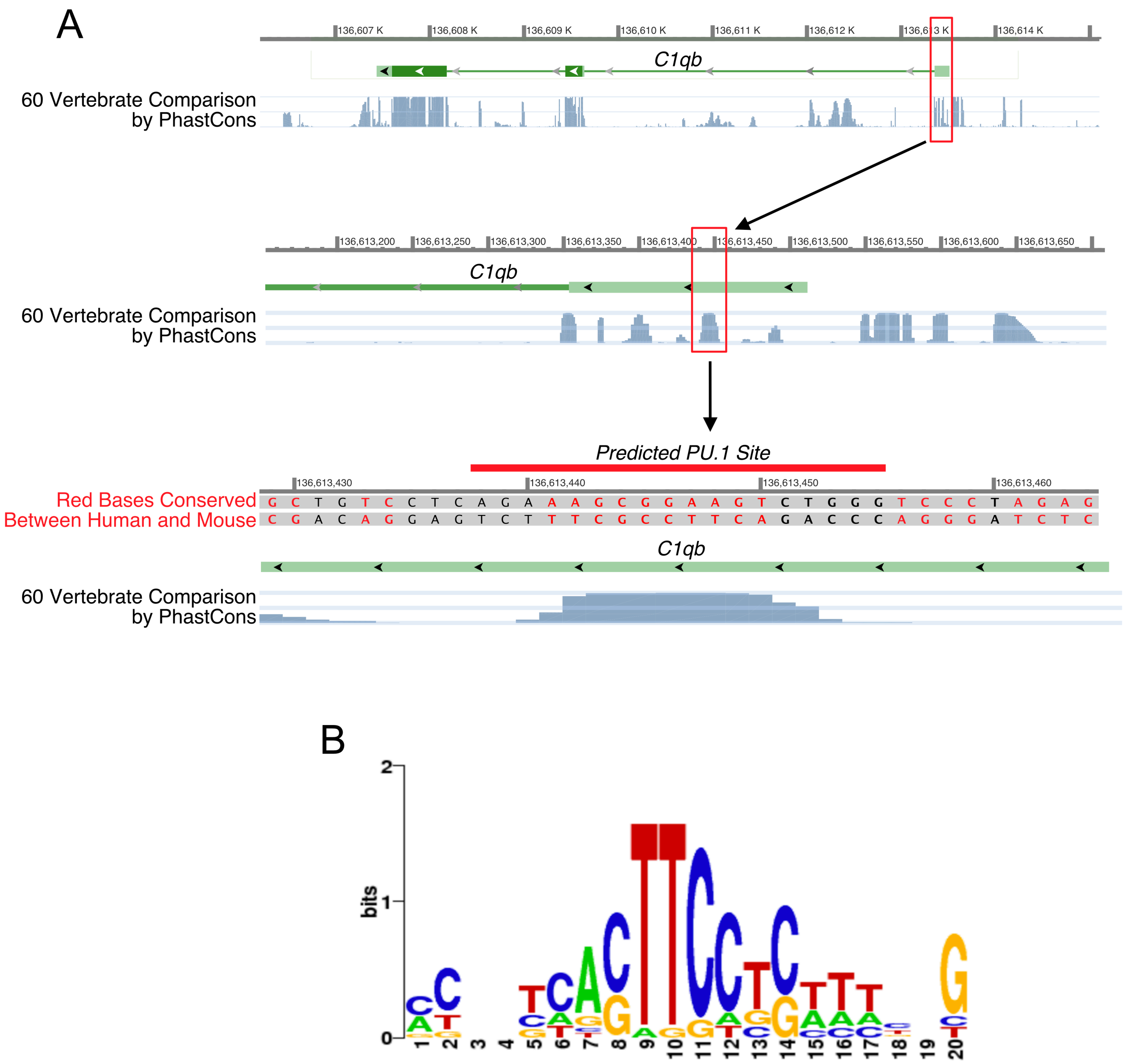

Supplemental Figure 8: C1q PU.1 consensus site is highly conserved across vertebrates. (A) Images from NCBI genome browser highlighting the C1q core promoter within the 5'UTR of the C1qb locus on mouse chromosome 4. Red boxes highlight subsequently magnified sequences. Red nucleotides are conserved between mouse and human. **(B)** PU.1 consensus site.

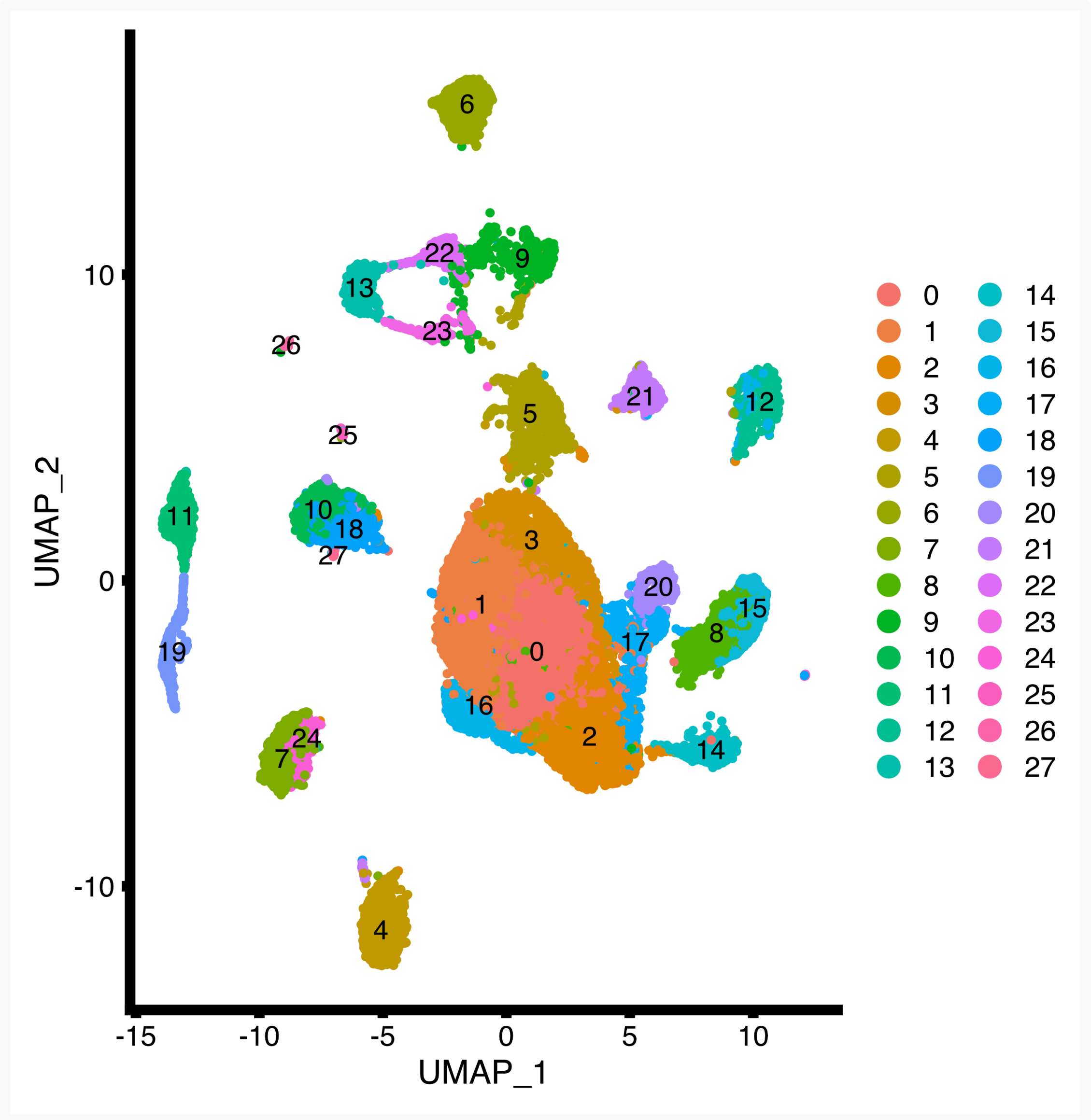

Supplemental Figure 9: UMAP of initial clustering of splenocytes. 26 clusters were identified.

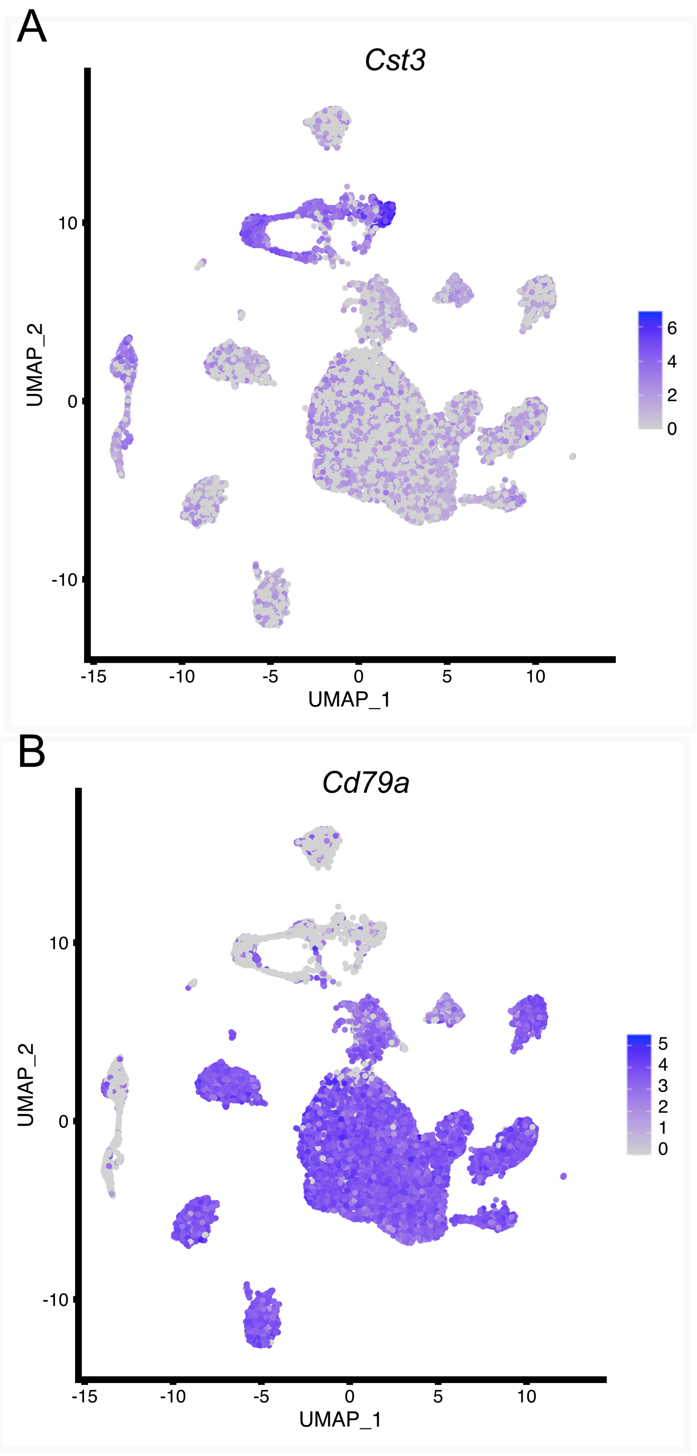

Supplemental Figure 10: Expression of *Cst3* and *Cd79a* on UMAP of initial clustering of splenocytes.

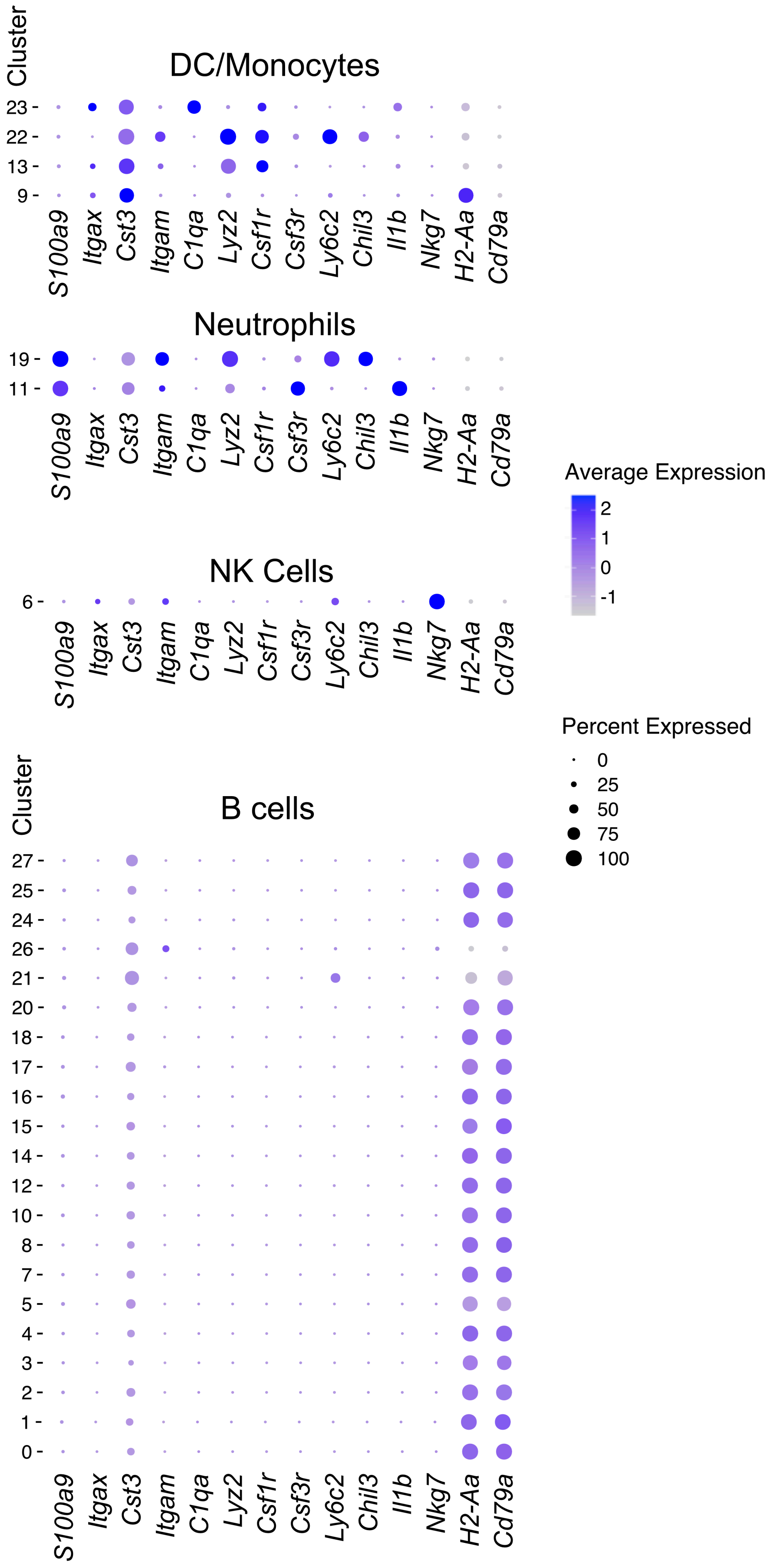

Supplemental Figure 11: Expression of cell-type specific genes in initial splenocyte clusters.

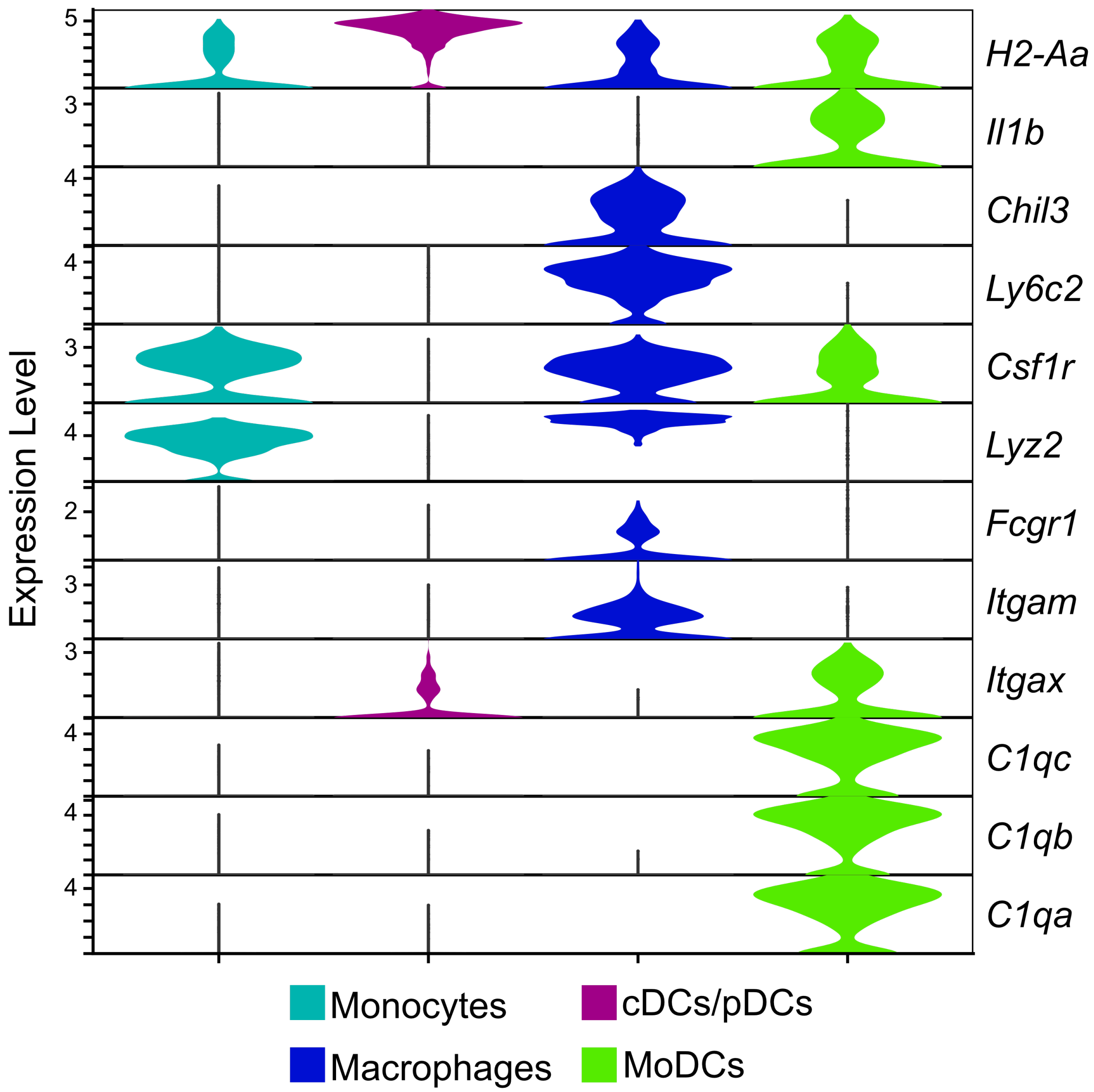

Supplemental Figure 12: Expression of selected genes to identify myeloid cell clusters.

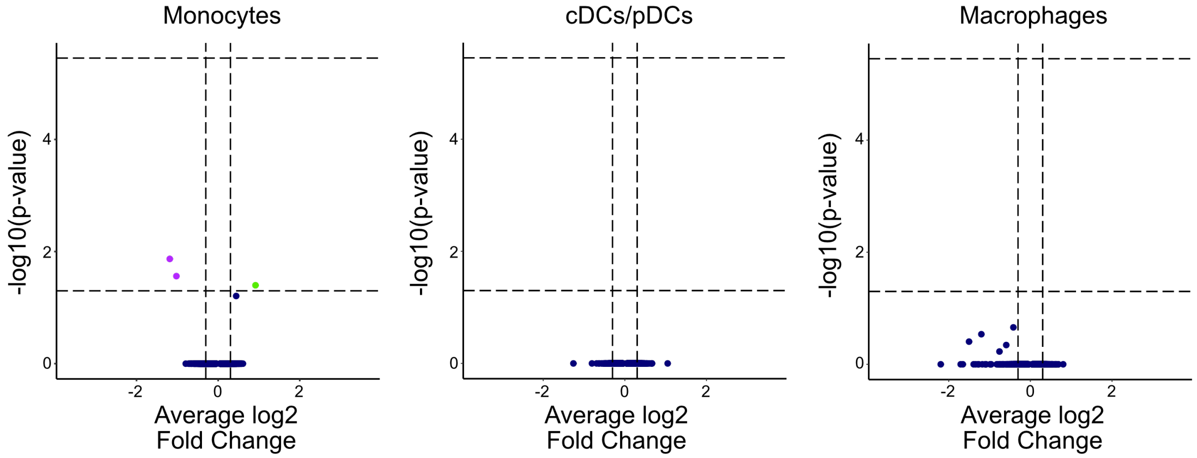

Supplemental Figure 13: Volcano plot identifying differentially expressed genes in the monocyte, cDC/pDC, and macrophage clusters between *B6.SLE123*+2HOBA and *B6.SLE123* mice.

	SLE Median [IQR] or # (%) (N = 11)	Control Median [IQR] or # (%) (N = 10)
Demographics		
Age, years	39 [27, 54]	34.5 [31, 64]
Race, # Caucasian	6 (55)	6 (60)
Ethnicity, # Hispanic	3 (27)	0 (0)
Sex, # female	10 (91)	7 (70)
Blood Pressure (Systolic)	123.5 [108-150]	129 [118-137]
Blood Pressure (Diastolic)	75 [51-93]	79 [63-89]
BMI	24.5 [17-40.2]	25.4 [22.2-36.7]
Creatinine	0.84 [0.76-0.87]	N/A
SLE Manifestation		
Mucocutaneous, # ever	9 (82)	N/A
Musculoskeletal, # ever	7 (64)	N/A
Serositis, # ever	8 (72)	N/A
Hematologic, # ever	6 (55)	N/A
Renal, # ever	6 (55)	N/A
Anti-dsDNA positivity, #	6 (60)	N/A
Low complement, # ever	6 (55)	N/A
SLEDAI, score	2 [2, 4]	N/A
SLICC, score	1 [0, 1]	N/A
Hypertension	2 (18)	0 (0)
Current medication use		
Hydroxycholoroquine, #	10 (91)	N/A
Mycophenolate, #	6 (55)	N/A
Corticosteroids, #	5 (45)	N/A
ACE Inhibitors	7 (64)	0 (0)

Supplementary Table 1: **Clinical features of SLE patients and controls studied for with flow cytometry.** Data on musculoskeletal involvement and anti-dsDNA antibody data unavailable in 1 subject. SLICC data unavailable in 2 subjects

	SLE Median [IQR] or # (%) (N = 6)	Control Median [IQR] .or # (%) (N = 4)
Demographics		
Age, years	29.5 [20, 36.75]	36.75 [29, 39]
Race, # Caucasian	5 (83)	4 (100)
Ethnicity, # Hispanic	1 (16.7)	1 (25)
Sex, # female	4 (67)	4 (100)
Blood Pressure (Systolic)	115.5 [105.5-127.75]	127.75 [126-129]
Blood Pressure (Diastolic)	70.5 [63.5-81.25]	73 [71-81.25]
BMI	22.1 [21.8-36.2]	26 [22.8-26.9]
Creatinine	0.71 [0.63-0.92]	N/A
SLE Manifestation		
Mucocutaneous, #	4 (66.7)	N/A
Musculoskeletal, #	5 (83.3)	N/A
Serositis, # ever	1 (16.7)	N/A
Hematologic, # ever	1 (16.7)	N/A
Renal, # ever	2 (33.3)	N/A
Anti-dsDNA positivity,	4 (66.7)	N/A
Low complement, #	2 (33)	N/A
SLEDAI, score	2 [1, 4]	N/A
SLICC, score	0 [0, 1]	N/A
Hypertension	0 (0)	0 (0)
Current medication		
Hydroxycholoroquine,	6 (100)	N/A
Mycophenolate, #	2 (33.3)	N/A
Corticosteroids, #	2 (33.3)	N/A
ACE Inhibitors	1 (16.7)	0 (0)

Supplemental Table 2: Clinical features of SLE patients and controls studies with mass spectrometry.

	SLE Median [IQR] or # (%) (N = 6)	Control Median [IQR] or # (%) (N = 4)
Demographics		
Age, years	46 [44, 50]	50 [36, 50]
Race, # Caucasian	4 (66.7)	4 (100)
Ethnicity, # Hispanic	0 (0)	0 (0)
Sex, # female	6 (100)	3 (75)
Blood Pressure (Systolic)	120.5 [111.25-123.75]	123.75 [100-125]
Blood Pressure	73 [66.75-74.75]	73 [57-75]
BMI	22.1 [21.8-36.2]	27.5 [25.9-30.7]
Creatinine	0.71 [0.63-0.92]	N/A
SLE Manifestation		
Mucocutaneous, #	4 (66.7)	N/A
Musculoskeletal, #	5 (83.3)	N/A
Serositis, # ever	1 (16.7)	N/A
Hematologic, # ever	1 (16.7)	N/A
Renal, # ever	2 (33.3)	N/A
Anti-dsDNA positivity,	4 (66.7)	N/A
Low complement, #	2 (33)	N/A
SLEDAI, score	2 [1, 4]	N/A
SLICC, score	0 [0, 1]	N/A
Hypertension	0 (0)	0 (0)
Current medication		
Hydroxycholoroquine,	6 (100)	N/A
Mycophenolate, #	2 (33.3)	N/A
Corticosteroids, #	2 (33.3)	N/A
ACE Inhibitors	1 (16.7)	0 (0)

Supplementary Table 3: Clinical features of SLE patients and controls studied with HPLC.

	SLE Median [IQR] or # (%) (N = 29)
Demographics	
Age, years	48.4 [40.9,58.5]
Race, # Caucasian	21 (72.4)
Ethnicity, # Hispanic	3 (10.3)
Sex, # female	28 (96.6)
Blood Pressure (Systolic)	120 [114-134]
Blood Pressure (Diastolic)	76 [70-85]
BMI	26.7 [22.9-33.8]
Creatinine	0.82 [0.77-0.91]
SLE Manifestation	
Mucosal ulcers, #	13 (44.8)
Myositis, #	2 (6.9)
Arthritis, #	6 (20.7)
Proteinuria, #	3 (10.3)
ANA, #	26 (89.7)
Low complement, #	7 (24.1)
SLEDAI, score	4 [2,8]

Supplementary Table 4: Clinical features of SLE patients and controls studied with anti-isoLG ELISA.

	Gene Name	BaseMean	pvalue	padj	FoldChange
ENSMUSG00000026399.12	*Cd55*	8.62E+02	7.26E-41	4.44E-39	3.95E-01
ENSMUSG00000024371.14	*C2*	3.70E+02	8.82E-34	3.56E-32	2.35E-01
ENSMUSG00000027082.15	*Tfpi*	1.54E+02	7.21E-24	1.50E-22	3.04E-01
ENSMUSG00000036905.8	*C1qb*	1.49E+04	2.23E-20	3.65E-19	2.10E-01
ENSMUSG00000036896.5	*C1qc*	1.54E+04	2.58E-20	4.19E-19	1.97E-01
ENSMUSG00000036887.5	*C1qa*	1.52E+04	6.01E-18	8.34E-17	2.09E-01
ENSMUSG00000055172.10	*C1ra*	1.10E+02	6.62E-18	9.13E-17	2.26E-01
ENSMUSG00000022181.16	*C6*	4.85E+03	1.74E-17	2.35E-16	1.80E-01
ENSMUSG00000044206.3	*Vsig4*	5.30E+01	1.01E-09	6.53E-09	2.61E-01
ENSMUSG00000021675.4	*F2rl2*	4.21E+01	1.94E-09	1.21E-08	2.61E-01
ENSMUSG00000037411.10	*Serpine1*	5.67E+01	5.94E-07	2.70E-06	3.54E-01
ENSMUSG00000040552.8	*C3ar1*	3.32E+01	2.06E-06	8.68E-06	3.71E-01
ENSMUSG00000032679.12	*Cd59a*	3.61E+01	6.91E-04	1.90E-03	4.89E-01
ENSMUSG00000026874.10	*Hc*	2.33E+01	3.64E-03	8.61E-03	2.05E+00

Supplementary Table 5: Complement and coagulation genes identified by RNA sequencing. Fold change is *B6.SLE123* compared to *C57BL6.*

Target	Antibody clone	Flourophore	Source	Panel
CD44	IM7	Phycoerythrin (PE)	Biolegend	B cell
CD45	30-F11	FITC	Biolegend	T cell
CD45	30-F11	PE-Cyanain-7 (Cy7)	Biolegend	B cell
CD45	30-F11	Pacific Orange	Thermo Fisher	Myeloid
CD3	17A2	PE-Cy7	Biolegend	T cell
CD4	RM4-5	Allophycocyanin (APC)	BD Biosciences	T cell
CD8	53-6.7	Amcyan	Biolegend	T cell
CD19	1D3	APC	Biolegend	B cell
CD138	281-2	Amcyan	BD Biosciences	B cell
F4/80	T45-2342	BV510	BD Biosciences	Myeloid
MerTK	108928	APC	R&D Systems	Myeloid
CD11b	M1/70	AF700	Biolegend	Myeloid
IAb	AF6-120.1	PE/Cy7	Biolegend	Myeloid
CD11c	N418	APC/Cy7	Biolegend	Myeloid
Viability		Zombie NIR	Biolegend	All cells

Supplementary Table 6: Antibodies and fluorophore tags used for flow cytometry.
